# Preclinical Evaluation of Baicalin for the Treatment of Diabetic Nephropathy: A Systematic Review and Meta-Analysis

**DOI:** 10.1055/a-2615-8249

**Published:** 2025-07-24

**Authors:** Hengtong An, Luyao Liu, Tongtong He, Xiaohan Chen, Xiaofei Jin, Xiaohong Zhou, Weijuan Gao

**Affiliations:** 1 College of Integrated Traditional Chinese and Western Medicine, Hebei University of Chinese Medicine, Shijiazhuang, Hebei, China; 2 Hebei Key Laboratory of Chinese Medicine Research on Cardio-Cerebrovascular Disease, Hebei University of Chinese Medicine, Shijiazhuang, Hebei, China

**Keywords:** *Scutellaria baicalensis*, Lamiaceae, baicalin, diabetic nephropathy, animal model, systematic review, mechanisms of action, meta-analysis

## Abstract

*Scutellaria baicalensis* , a widely used medicinal herb in traditional Chinese medicine, is frequently employed in the treatment of diabetic nephropathy (DN). Its primary active constituent, baicalin, has shown significant therapeutic potential in animal models of DN; however, no comprehensive and systematic evaluation of its therapeutic effects and underlying mechanisms in DN has yet been conducted. This meta-analysis aimed to assess the efficacy of baicalin in DN treatment and elucidate its pharmacological mechanisms. Relevant studies were retrieved from databases including Web of Science, PubMed, Embase, CNKI, Wanfang Data, and VPCS, covering the literature up to November 2024. Study quality was evaluated using SYRCLEʼs risk of bias tool, and statistical analyses were performed with STATA 12. Primary outcomes included blood urea nitrogen (BUN), serum creatinine (SCR), and fasting blood glucose (FBG), while secondary outcomes encompassed urinary protein (UP), triglycerides (TG), total cholesterol (TC), inflammatory markers, fibrosis indicators, and oxidative stress parameters. Subgroup analyses, publication bias assessments, and sensitivity analyses were conducted to ensure result reliability. A total of 14 studies involving 221 rodents met the inclusion criteria. Baicalin significantly reduced BUN, SCR, FBG, TG, TC, UP, interleukin-6 (IL-6), interleukin-1 beta (IL-1 *β* ), tumor necrosis factor-alpha (TNF- *α* ), malondialdehyde (MDA), and fibronectin (FN) levels while enhancing superoxide dismutase (SOD) activity. These findings suggest that baicalin improves kidney function, reduces proteinuria, corrects lipid metabolism, and alleviates inflammation, oxidative stress, and fibrosis. This meta-analysis concludes that baicalin exhibits significant therapeutic potential in DN models, acting via anti-inflammatory, antioxidant, and antifibrotic mechanisms.

**Abbreviations**
ACEIs: angiotensin-converting enzyme inhibitors
ADA: American Diabetes Association
AGEs: advanced glycation end products
ARBs: angiotensin II receptor blockers
Bax: Bcl-2 Associated X Protein
Bcl-2: B-cell Lymphoma/Leukemia 2
BUN: blood urea nitrogen
Caspase-9: Cysteine Aspartic Acid Specific Proteinase 9
CAT: Catalase
CIs: confidence intervals
CKD: chronic kidney disease
COL-Ⅰ: Collagen Type I
COL-Ⅲ: Collagen Type Ⅲ
COL-Ⅳ: Collagen IV
CPT1A: Carnitine palmitoyltransferase 1A
DAMPs: danger-associated molecular patterns
DM: diabetes mellitus
DN: diabetic nephropathy
ESRD: end-stage renal disease
FAO: Fatty acid oxidation
FBG: fasting blood glucose
FN: Fibronectin
GLP-1: glucagon-like peptide-1
GSH-Px: glutathione peroxidase
H2O2: hydrogen peroxide
I²: I-squared
IL-1 *β*: interleukin-1 beta
IL-6: interleukin-6
MDA: malondialdehyde
Myd88: Myeloid Differentiation Primary Response 88
NLRP3: NOD-like receptor family pyrin domain containing 3
NO: nitric oxide
NOX: NADPH oxidase
Nrf2: nuclear factor erythroid 2-related factor 2
PAMPs: Pathogen-associated molecular patterns
PKC: protein kinase C
RAGE: alongside their receptor
SCR: serum creatinine
SGLT2: sodium-glucose cotransporter 2
SMDs: standardized mean differences
SOD: superoxide dismutase
TC: total cholesterol
TECs: tubular epithelial cells
TG: triglycerides
TLR4: Toll-like Receptor 4
TLRs: Toll-like receptors
TNF- *α*: tumor necrosis factor-alpha
UP: urinary protein
WT1: Wilms’ tumor 1 gene
*α* SMA
: *α* -Smooth Muscle Actin
 

## Introduction

Diabetic nephropathy (DN) is recognized as a prominent microvascular complication associated with diabetes mellitus (DM), clinically marked by the presence of proteinuria, swelling, high blood pressure, and a gradual deterioration of kidney function. In its advanced phases, DN can evolve into end-stage renal disease (ESRD) 1 , significantly diminishing the quality of life for patients. The 10th edition of the *IDF Diabetes Atlas* 2 estimates that in 2021, around 510 million adults worldwide had diabetes, with projections suggesting this figure could escalate to 640 million by 2045. DN poses a formidable challenge within modern medical treatment paradigms. Although the American Diabetes Association (ADA) recommends several medications–such as angiotensin-converting enzyme inhibitors (ACEIs), angiotensin II receptor blockers (ARBs), sodium-glucose cotransporter 2 (SGLT2) inhibitors, and glucagon-like peptide-1 (GLP-1) receptor agonists–which have been integrated into clinical practice, DN continues to be a primary contributor to ESRD 3 . Additionally, DN has emerged as a key risk factor for cardiovascular and cerebrovascular diseases 4 , constituting a considerable threat to public health as a dominant chronic non-communicable disease. This situation imposes significant economic burdens on individuals, families, and society at large. Consequently, understanding the pathogenesis of DN and developing effective strategies and pharmacological interventions to decelerate the progression of DN are primary research objectives in nephrology today, carrying substantial clinical importance.

**Fig. 1:**
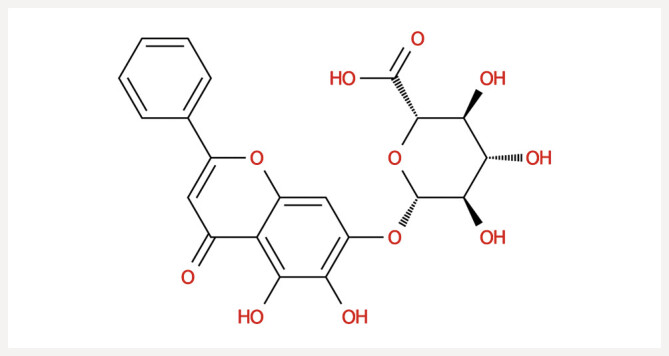
The chemical structure of baicalin.

*Scutellaria baicalensis* Georgi ( *Lamiaceae* ), known in traditional Chinese medicine as Huangqin, is extensively utilized for its therapeutic properties. Baicalin, a flavonoid derivative extracted from Huangqin, is defined by its structure, which consists of a flavone backbone (flavonoid glycoside) attached to multiple sugar units, with the molecular formula C21H18O11 ( Fig. 1 ). Investigations into its pharmacological attributes have revealed that baicalin not only modulates inflammatory pathways and decreases the production of various pro-inflammatory cytokines 5 but also neutralizes free radicals, thus reducing oxidative stress 6 . Moreover, baicalin has demonstrated the ability to inhibit cancer cell proliferation and trigger programmed cell death in these cells 7 . Numerous studies have validated baicalinʼs protective effects against DN in animal models through distinct mechanisms 6 , 8 .

At present, research on baicalin primarily exists within the realm of preclinical studies. Despite substantial evidence suggesting that baicalin has beneficial effects on DN, these outcomes are derived from experiments across various laboratories, resulting in inconsistent findings. Thus, employing a unified methodology to integrate these outcomes is crucial. A systematic review serves as a type of secondary research, synthesizing findings from studies that address specific research questions. This technique allows for pooling data from multiple studies, thereby increasing sample sizes and enhancing the statistical power. Furthermore, by systematically including research and applying stringent methods to evaluate and control for biases, the influence of biases can be mitigated while also minimizing unnecessary animal use. Developing evidence-based experiments at the animal level facilitates the translation of preclinical findings into clinical applications. As of now, there has been no exhaustive summary of high-quality evidence-based medical data nor an analysis of the pharmacological pathways through which baicalin impacts DN. In this study, we aim to clarify the renal protective effects of baicalin in DN animal models and elucidate its underlying mechanisms by compiling pertinent preclinical research data and performing a systematic review along with a meta-analysis.

## Methods

### Systematic review registration

The systematic review and meta-analysis were conducted in accordance with PRISMA guidelines and have been registered in the INPLASY database. This process ensures transparency and rigor in the methodology adopted for the research undertaken (Registration No. INPLASY2024120099).

### Data sources and search strategy

**Fig. 2:**
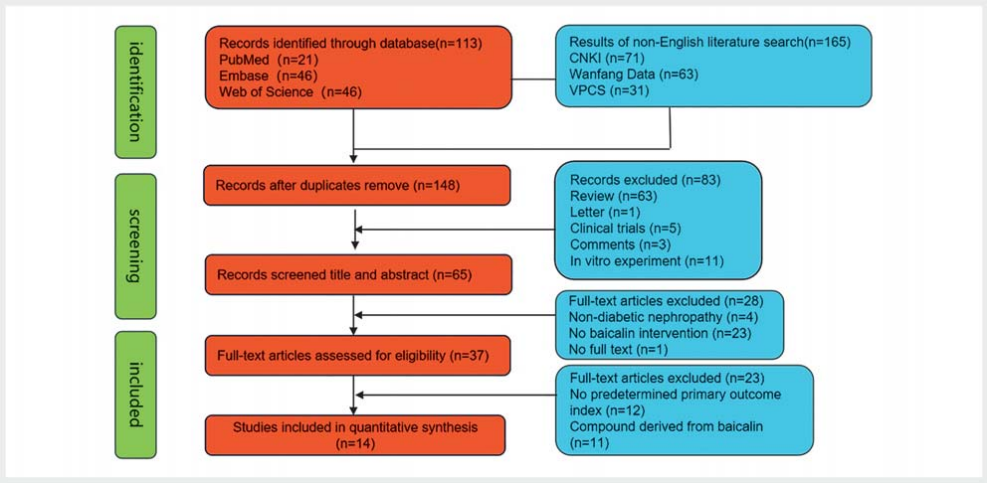
Selection of studies for the meta-analysis.

To enhance the comprehensiveness and reliability of this meta-analysis, literature searches were conducted in both English and Chinese databases to identify relevant preclinical studies. English databases included PubMed, Web of Science, and Embase, while Chinese databases included China National Knowledge Infrastructure (CNKI), Wanfang Data, and the VIP Database for Chinese Technical Periodicals (VPCS). All available literature published up to September 2024 was considered. A combination of subject headings and free-text terms was employed using keywords such as “baicalin”, “Diabetic Nephropathies”, “Nephropathies, Diabetic”, or “Nephropathy, Diabetic”, “Diabetic Kidney Disease”, “Diabetic Kidney Diseases”, “Kidney Disease, Diabetic”, “Kidney Diseases, Diabetic”, “Diabetic Nephropathy”, “Diabetic Glomerulosclerosis”, “Glomerulosclerosis, Diabetic”, “Intracapillary Glomerulosclerosis”, “Kimmelstiel-Wilson Disease”, “Kimmelstiel Wilson Disease”, “Nodular Glomerulosclerosis”, “Glomerulosclerosis, Nodular”, “Kimmelstiel-Wilson Syndrome”, “Kimmelstiel Wilson Syndrome”, and “Syndrome, Kimmelstiel-Wilson”. The detailed search strategy is presented in Fig. 2 and Supplementary Materials.

### Inclusion and exclusion criteria

The identification of pertinent studies was guided by inclusion criteria established in accordance with the PICO framework: (1) involvement of animals modeled for DN; (2) administration of baicalin in varying dosages within the treatment cohort; (3) the existence of a control group that was administered either a placebo or no intervention; and (4) primary outcomes measured as blood urea nitrogen (BUN), serum creatinine (SCR), and fasting blood glucose (FBG), with secondary outcomes including urinary protein (UP), triglycerides (TG), and total cholesterol (TC), alongside markers of inflammation, oxidative stress, and fibrosis. The criteria for exclusion were established as follows: (1) exclusion of non-animal studies, such as clinical trials, case reports, reviews, and cellular experiments; (2) studies aligning with inclusion criteria yet lacking available full texts; (3) lack of a separate control group or studies including multi-drug regimens; (4) duplicate publications; and (5) absence of crucial data like sample size, standard deviation, or standard error in the studies.

### Study selection and data extraction

Two independent reviewers (Hengtong An and Luyao Liu) performed the literature search, study selection, data extraction, and quality assessment according to pre-specified inclusion and exclusion criteria. Any disagreements were resolved through discussion or consultation with the corresponding author.

For data extraction, the same two reviewers independently collected the following information: (1) publication year and first authorʼs name; (2) detailed descriptions of experimental animals, including species, sex, age, and weight; (3) methods of diabetic model induction, specifying administration routes, dosages, modeling duration, and diagnostic criteria; (4) intervention strategies for both experimental and control groups, including administration methods, dosages, and treatment durations; (5) primary and secondary outcome measures. When multiple drug concentrations were reported, the highest was selected. For outcomes reported at different time points, the most recent data before euthanasia was used.

For studies where outcomes were available only in graphical format, attempts were made to contact the original authors to obtain raw data. In the absence of a response, data were extracted using WebPlotDigitizer4.5. To ensure accuracy, the same two reviewers independently digitized the graphs and performed cross-checking. Discrepancies during data extraction were resolved by discussion or in consultation with the corresponding author. Data presented as SEM were converted to SD using the formula SD = SEM × √n 9 .

### Risk of bias

A risk assessment was independently performed by researchers utilizing the SYRCLEʼs risk of bias tool, which is supplied by the Animal Intervention Research Centre, specifically for animal studies. This tool comprises 10 items designed to assess potential bias risk 9 . The specific items are as follows: (1) sequence generation, (2) baseline characteristics, (3) allocation concealment, (4) random housing, (5) blinding (performance bias), (6) random outcome assessment, (7) blinding (detection bias), (8) incomplete outcome data, (9) selective outcome reporting, and (10) other sources of bias (each item scores 1 point, for a total of 10 points). Any inconsistencies that emerged during the risk assessment were addressed by consulting with the corresponding author.

### Subgroup analysis

Four subgroups were predetermined for our analysis: (1) animal species, (2) methods of modeling, (3) duration of intervention, and (4) dosage regimens of baicalin. Subgroup analyses were performed on the primary outcomes to assess how these variables or study characteristics influenced the estimated effect sizes. Furthermore, these analyses served to explore the underlying sources of heterogeneity when significant heterogeneity was observed.

### Statistical analysis

Statistical analyses were conducted using Stata 12. The dataset comprised continuous variables, with overall effect sizes represented as standardized mean differences (SMDs) along with 95% confidence intervals (CIs). The I-squared (I²) statistic served to assess heterogeneity; an I² value greater than 50% was considered indicative of significant heterogeneity, resulting in the selection of a random effects model, whereas a fixed effects model was utilized in alternative scenarios. Statistical significance was determined by P < 0.05.

### Publication bias

Evaluation of publication bias related to the primary outcome measures was carried out through funnel plots and Eggerʼs test. The statistical analysis was executed using Stata version 12. Funnel plots were produced with the command “db metafunnel”, and Eggerʼs test was applied using the command “db metabias _ES _seES, graph(egger)”. P < − 0.05 was considered indicative of statistical significance. When substantial bias risk was identified, the trim-and-fill method was utilized to adjust the data from the included studies, thereby calculating a combined estimate of the overall effect. The applicable commands for the trim-and-fill approach were “metatrim _ES _seES, flip funnel”. This methodology helped assess the stability of results in studies where bias risk was detected.

### Sensitivity analysis

Irrespective of the subgroup analysisʼs capability to pinpoint the origins of heterogeneity, conducting a sensitivity analysis is essential for aiding researchers in evaluating the robustness of the outcomes derived from the meta-analysis. This was executed through the application of the “db metaninf” command. P < 0.05 was deemed statistically significant.

### Dose–time–response analysis

To evaluate the effects of different intervention durations and dosing regimens of baicalin on diabetic nephropathy-related outcomes, time–dose–response plots were generated for the primary endpoints–BUN, SCR, and FBG–to visualize the relationship between baicalin dosage, treatment duration, and therapeutic efficacy.

## Results

### Study selection

We retrieved a total of 278 articles from the database, including 21 from PubMed, 46 from Embase, 46 from Web of Science, 71 from CNKI, 63 from Wanfang Data, and 31 from VPCS. After merging all search results and removing duplicates, 148 articles were retained. Subsequently, we excluded 83 articles by reviewing their titles and abstracts for the following reasons: (1) reviews, (2) letters, (3) consensus statements, (4) clinical trials, and (5) *in vitro* experiments. Then, after conducting a full-text review of the remaining articles, we excluded an additional 37 articles for the following reasons: (1) non-diabetic nephropathy models, (2) lack of intervention with baicalin, (3) combined interventions, and (4) absence of predefined outcome measures. Ultimately, 14 articles that met the criteria were included (as shown in Fig. 2 ).

### Characteristics of included studies

(1) The included studies were published within the last five years (2007 – 2024). (2) A total of 14 studies included 221 animal models of diabetic nephropathy. Among these, 4 studies used db/db mice (60/221, 27.15%), 4 studies utilized Sprague–Dawley rats (77/221, 34.84%), 3 studies employed Wistar rats (48/221, 20.36%), 2 studies used C57BL/6j mice (24/221, 15.69%), and 1 study used Kunming mice (12/221, 5.43%). (3) In the 14 studies, 11 studies involved male experimental animals, 1 study involved female animals, while 2 studies did not specify the sex. (4) Ten studies mentioned the ages of the experimental animals, while four did not specify; nine studies reported the body weights of the experimental animals, and five did not provide this information. (5) For the construction of the diabetic model, 10 studies used STZ induction, and 4 studies utilized spontaneous DM models. (6) Regarding the criteria for successful modeling, excluding spontaneous DM models, 7 studies defined the modeling standard as FBG > 16.7 mmol/L, while 3 studies used FBG > 16 mmol/L as the standard. (7) The duration of administration ranged from a minimum of 4 weeks to a maximum of 16 weeks. (8) The minimum concentration of baicalin was 40 mg/kg, while the maximum concentration was 400 mg/kg. (9) Regarding the routes of administration, 12 studies employed oral gavage, while 2 studies used intraperitoneal injection. (10) Concerning the outcome measures, 9 studies evaluated BUN levels, 9 studies assessed SCR levels, 9 studies reported FBG levels, 7 studies noted UP levels, and 6 studies measured TG and TC levels. In addition, some studies reported inflammatory markers such as Interleukin-6 (IL-6) and Interleukin-1 *β* (IL-1 *β* ). Furthermore, some studies provided data on antioxidant markers such as superoxide dismutase (SOD) and malondialdehyde (MDA), while others recorded fibrosis-related markers such as fibronectin (FN). For further details, please refer to Table 1 .

**Table:** **Table 1** Basic characteristics of the included studies.

Study (Year)	Species (Sex; n = treatment/control group), Weight	Model (establish; modeling standard)	Treatment group (baicalin) (administration; dose; course of treatment)	Outcome Index	Intergroup Differences
Hu SH et al. (2007) 16	Wistar rats (male, 8/6), 180 – 200 g	Intraperitoneal injection of STZ (60 mg/kg), BG > 16.67 mmol/L	By Intragastric, 200 mg/kg/d, 4 weeks	1. FBG	P > 0.05
				2. HbA1c	P < 0.01
Su N et al. (2007) 12	SD rats (male, 10/10), 230 – 300 g	Intraperitoneal injection of STZ (55 mg/kg), BG > 16 mmol/L	By intraperitoneal injection, 40 mg/kg/d, 6 weeks	1. UP	P < 0.05
				2. SOD	P < 0.05
				3. GSH-PX	P < 0.05
Liu CS et al. (2007) 11	Wistar rats (male, 10/9), 180 – 200 g	Intraperitoneal injection of STZ (60 mg/kg), BG > 16.7 mmol/L	By Intragastric, 150 mg/kg/d, 16 weeks	1. FBG	P < 0.01
				2. AR	P < 0.01
Sun J et al. (2019) 10	Wistar rats (male, 8/7), 200 – 250 g	Intraperitoneal injection of STZ (30 mg/kg), BG > 16.7 mmol/L	By Intragastric, 150 mg/kg/d, 6 weeks	1. FBG	P < 0.05
				2. TC	P < 0.05
				3. TG	P < 0.05
				4. Survivin	P < 0.05
Zhang SF et al. (2020) 21	C57 mice (male, 6/6), 20 – 25 g	Intraperitoneal injection of STZ (125 mg/kg), BG > 16 mmol/L	By Intragastric, 45 mg/kg/d, 8 weeks	1. BUN	P < 0.05
				2. SCR	P < 0.05
				3. FN	P < 0.05
				4. COL-Ⅳ	P < 0.05
Zhang XT et al. (2020) 18	Kunming mice (Female, 6/6), NR	Intraperitoneal injection of STZ (75 mg/kg), BG > 16 mmol/L	By Intragastric, 40 mg/kg/d, 12 weeks	1. BUN	P < 0.05
				2. SCR	P < 0.05
				3. FBG	P < 0.001
				4. MDA	P < 0.01
				5. SOD	P < 0.001
				6. Nephrin	P < 0.05
				7. WT1	P < 0.001
				8. Caspase-9	P < 0.001
				9. PCNA	P < 0.01
Zheng XP et al. (2020) 17	SD rats (male, 10/15), 180 – 200 g	Intraperitoneal injection of STZ (65 mg/kg), BG > 16.7 mmol/L	By Intragastric, 160 mg/kg/d, 8 weeks	1. BUN	P < 0.01
				2. SCR	P < 0.01
				3. FBG	P < 0.01
				4. UP	P < 0.01
				5. TC	P < 0.01
				6. TG	P < 0.01
				7. p65	P < 0.01
				8. IL-1 *β*	P < 0.05
				9. IL-6	P < 0.05
				10. TGF-1/Smad3	P < 0.01
				11. FN	P < 0.01
Ma LY et al. (2021) 6	db/db mice (male, 8/8), NR	Spontaneous diabetic model	By Intragastric, 400 mg/kg/d, 8 weeks	1. BUN	P > 0.05
				2. SCR	P > 0.05
				3. FBG	P < 0.05
				4. Caspase-3	P < 0.001
				5. Bax	P < 0.001
				6. Bcl-2	P < 0.001
				7. IL-1 *β*	P < 0.05
				8. IL-6	P < 0.001
				9. TNF- *α*	P < 0.05
				10. SOD	P < 0.05
				11. CAT	P < 0.05
				12. MDA	P < 0.05
				13. Nrf2/MAPK	P < 0.001
Ou Y et al. (2021) 14	SD rats (male, 6/6), 180 – 200 g	Intraperitoneal injection of STZ (35 mg/kg), BG > 16.7 mmol/L	By Intragastric, 200 mg/kg/d, 4 weeks	1. BUN	P < 0.01
				2. SCR	P < 0.01
				3. FBG	P < 0.01
				4. UP	P < 0.01
				5. TC	P < 0.01
				6. TG	P < 0.01
				7. TNF- *α*	P < 0.001
				8. IL-1 *β*	P < 0.001
				9. IL − 6	P < 0.001
				10. LDH	P < 0.001
				11. SOD	P < 0.01
				12. MDA	P < 0.001
				13. PI3K/Akt/mTOR	P < 0.01
Wang BY et al. (2021) 22	C57 mice (male, 6/6), 25 g	Intraperitoneal injection of STZ (50 mg/kg), BG > 16.7 mmol/L	By Intragastric, 80 mg/kg/d, 12 weeks	1. BUN	P < 0.05
				2. SCR	P < 0.05
				3. UP	P < 0.05
				4. IL-6	P < 0.05
				5. TNF- *α*	P < 0.05
				6. IL-1 *β*	P < 0.05
				7. NF- *κ* B	P < 0.05
				8. TLR4	P < 0.05
				9. Myd88	P < 0.05
Zhang Y et al. (2022) 20	db/db mice (male, 6/6), NR	Spontaneous diabetic model	By Intragastric, 100 mg/kg/d, 8 weeks	1. BUN	P > 0.05
				2. SCR	P > 0.05
				3. TC	P > 0.05
				4. TG	P < 0.01
				5. SIRT1/AMPK/HNF4A	P < 0.05
Ren GF et al. (2023) 13	db/db mice (male, 10/10), NR	Spontaneous diabetic model	By Intragastric, 100 mg/kg/d, 12 weeks	1. BUN	P < 0.001
				2. SCR	P < 0.001
				3. FBG	P < 0.001
				4. UP	P < 0.001
				5. TC	P < 0.001
				6. TG	P < 0.001
				7. TNF- *α*	P < 0.001
				8. IL-1 *β*	P < 0.001
				9. IL − 6	P < 0.001
				10. CAT	P < 0.001
				11. MDA	P < 0.001
				12. SOD	P < 0.001
				13. SphK1/S1P/NF- *κ* B	P < 0.05
Zhang XD et al. (2023) 15	SD rats (male, 10/10), 200 – 220	Intraperitoneal injection of STZ (65 mg/kg), NR	By Intragastric, 150 mg/kg/d, 4 weeks	1. BUN	P < 0.01
				2. SCR	P < 0.01
				3. FBG	P < 0.05
				4. UP	P < 0.01
				5. TLR4/MAPK/NF- *κ* B	P < 0.01
				6. COL-Ⅰ	P < 0.01
				7. COL-Ⅲ	P < 0.01
Hu HT et al. (2024) 19	db/db mice (male, 6/6), NR	Spontaneous diabetic model	By Intragastric, 50 mg/kg/d, 12 weeks	1. FBG	P > 0.05
				2. UP	P > 0.05
				3. TC	P < 0.05
				4. TG	P < 0.05
				5. TNF- *α*	P < 0.05
				6. IL-1 *β*	P < 0.05
				7. IL − 6	P < 0.05
				8. *α* SMA	P < 0.001
				9. FN	P < 0.05
BUN: Blood Urea Nitrogen; SCR: Serum Creatinine; FN: Fibronectin; COL-Ⅳ: Collagen IV; FBG: Fasting Blood Glucose; HbA1c: Glycated Hemoglobin; MDA: Malondialdehyde; SOD: Superoxide Dismutase; WT1: Wilmsʼ tumor 1 gene; caspase-9: Cysteine Aspartic Acid Specific Proteinase 9; Bax: Bcl-2 Associated X Protein; Bcl-2: B-cell Lymphoma/Leukemia 2; IL-1 *β* : Interleukin-1 beta; IL-6: Interleukin-6; TNF- *α* : Tumor Necrosis Factor-alpha; SOD: Superoxide Dismutase; CAT: Catalase; UP: Urinary Protein; TLR4: Toll-like Receptor 4; Myd88: Myeloid Differentiation Primary Response 88; TC: Total Cholesterol; TG: Triglycerides; COL-Ⅰ: Collagen Type I; COL-Ⅲ: Collagen Type Ⅲ; *α* SMA: *α* -Smooth Muscle Actin					

### Study quality

**Fig. 3:**
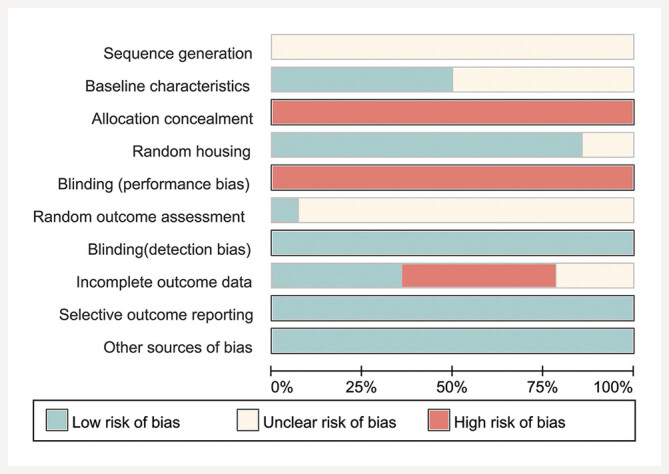
Risk of bias graph.

As shown in Fig. 3 and Supplementary **Figure 1** , we evaluated the quality of all the studies included in this research. The scores of each study ranged from 4 to 6 points; 6 studies received a score of 4 6 , 10 , 11 , 12 , 13 , 14 , 5 studies received a score of 5 15 , 16 , 17 , 18 , 19 , and 3 studies received a score of 6 20 , 21 , 22 . All 14 studies reported random allocation, but none explicitly described the randomization method used. Seven studies failed to report how baseline characteristics were balanced. No study reported on the concealment of allocation. Twelve studies documented the randomization of animal placement, while two study did not report this information. None of the studies indicated that blinding was implemented for the experimenters. Nine studies did not include all experimental animals in the outcome analysis. All 14 studies reported results in accordance with the experimental protocols. There were no other sources of bias identified in any of the studies.

## Effectiveness

### Primary outcomes

**Fig. 4:**
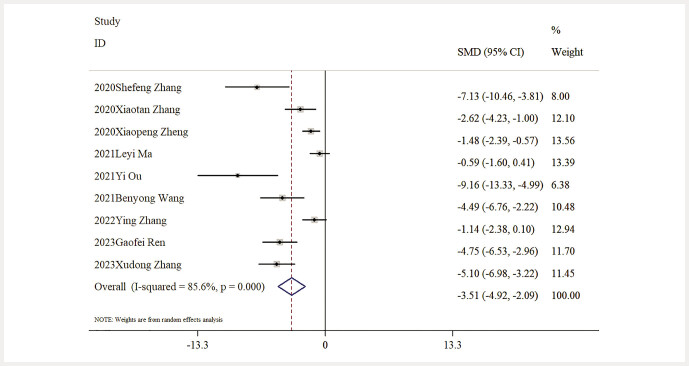
Forest plot: impact of baicalin on BUN level 6 , 13 , 14 , 15 , 17 , 18 , 20 , 21 , 22 .

Impact of Baicalin on BUN Levels. Data concerning BUN levels were provided by nine studies. An extensive analysis indicated that baicalin led to a significant decrease in BUN levels when compared to the model group [n = 141, SMD: − 3.51 (95% CI: − 4.92, − 2.10), P < 0.05; heterogeneity: I² = 85.6%, P < 0.01; Fig. 4 .

**Fig. 5:**
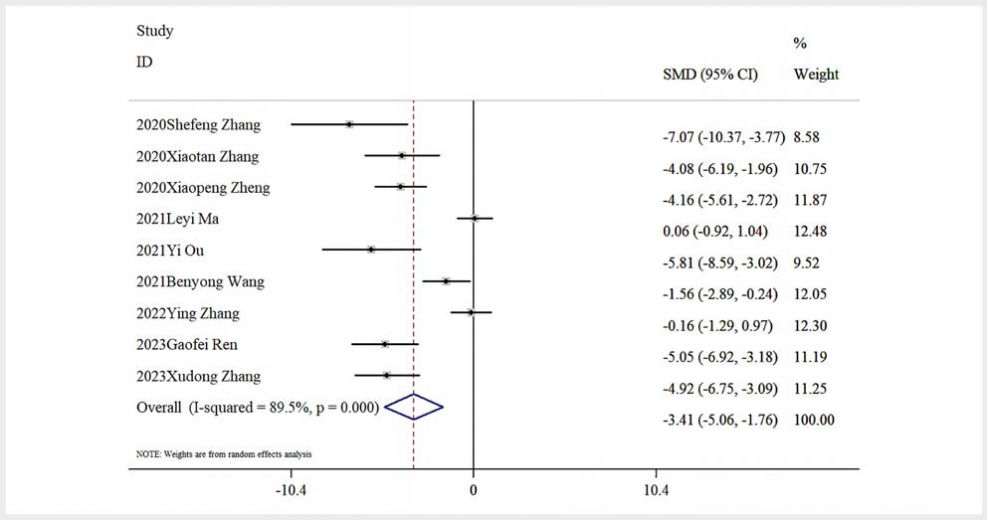
Forest plot: impact of Baicalin on SCR level 6 , 13 , 14 , 15 , 17 , 18 , 20 , 21 , 22 .

Impact of Baicalin on SCR Levels. Nine studies contributed data regarding SCR levels. The detailed analysis demonstrated that baicalin resulted in a significant reduction in SCR levels in comparison to the model group [n = 141, SMD: − 3.41 (95% CI: − 5.06, − 1.76), P < 0.05; heterogeneity: I² = 89.5%, P < 0.01; Fig. 5 .

**Fig. 6:**
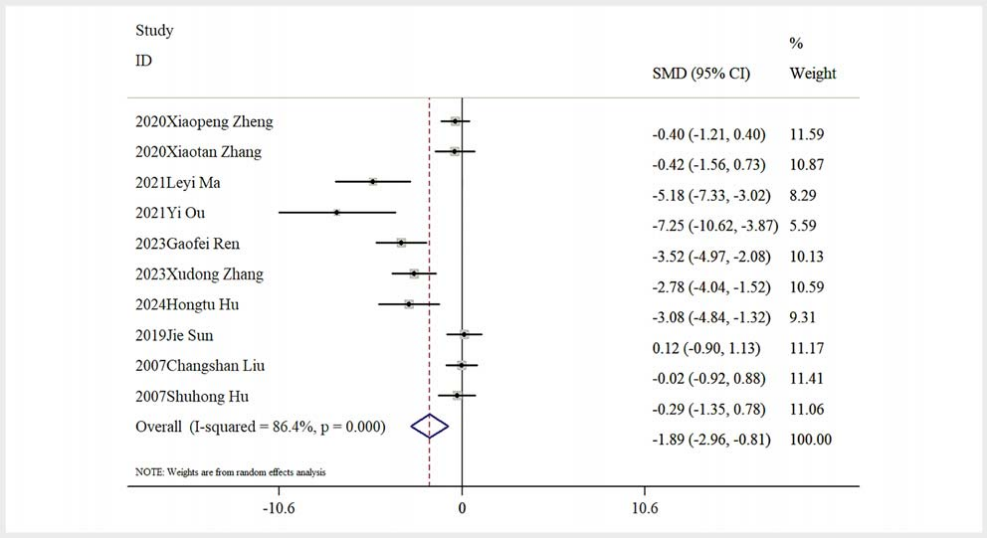
Forest plot: impact of baicalin on FBG level 6 , 10 , 11 , 13 , 14 , 15 , 16 , 17 , 18 , 19 .

Impact of Baicalin on FBG Levels. Data on FBG levels were available from nine studies. The thorough analysis revealed that baicalin significantly lowered FBG levels relative to the model group [n = 165, SMD: − 1.89 (95% CI: − 2.96, − 0.81), P < 0.05; heterogeneity: I² = 86.4%, P < 0.01; Fig. 6 .

### Secondary outcomes

**Fig. 7:**
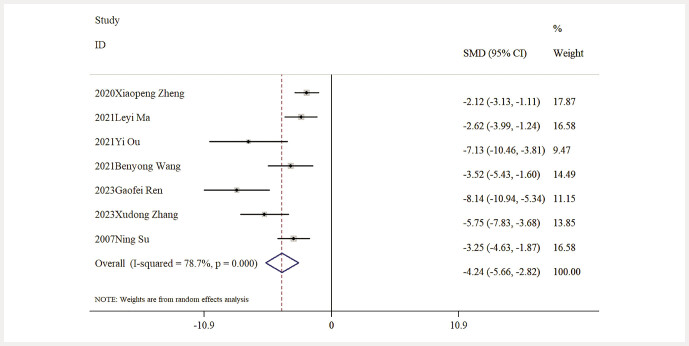
Forest plot: impact of baicalin on UP level 6 , 12 , 13 , 14 , 15 , 17 , 22 .

Influence of Baicalin on UP Levels. Seven studies provided data regarding UP levels. A thorough analysis demonstrated that baicalin significantly decreased UP levels in comparison to the model group [n = 125, SMD: − 4.24 (95% CI: − 5.66, -2.82), P < 0.05; heterogeneity: I² = 78.7%, P < 0.01; Fig. 7 .

**Fig. 8:**
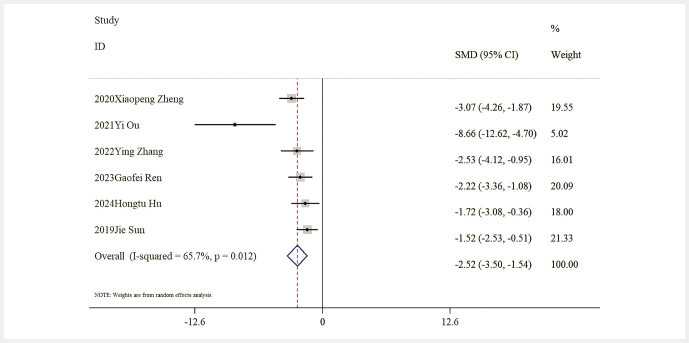
Forest plot: impact of baicalin on TG level 10 , 13 , 14 , 17 , 19 , 20 .

**Fig. 9:**
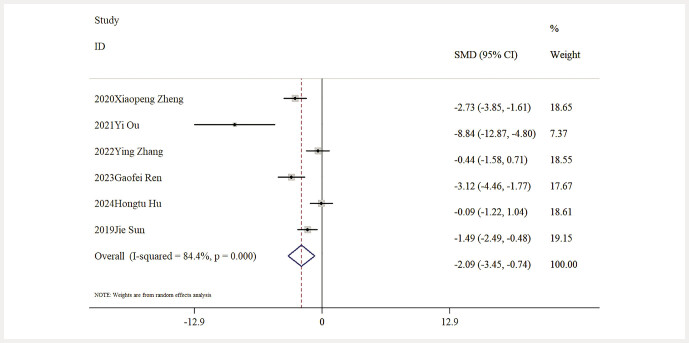
Forest plot: impact of baicalin on TC level 10 , 13 , 14 , 17 , 19 , 20 .

Influence of Baicalin on Lipid Metabolism. Six studies reported data on TG levels, indicating that baicalin significantly reduced TG levels compared to the model group [n = 101, SMD: − 2.52 (95% CI: − 3.5, − 1.54), P < 0.05; heterogeneity: I² = 65.7%, P = 0.012; Fig. 8 . Additionally, data on TC levels were reported by six studies. The findings of the comprehensive analysis revealed that baicalin led to a significant decrease in TC levels when compared to the model group [n = 101, SMD: − 2.09 (95% CI: − 3.45, − 0.74), P < 0.05; heterogeneity: I² = 84.4%, P < 0.01; Fig. 9 .

**Fig. 10:**
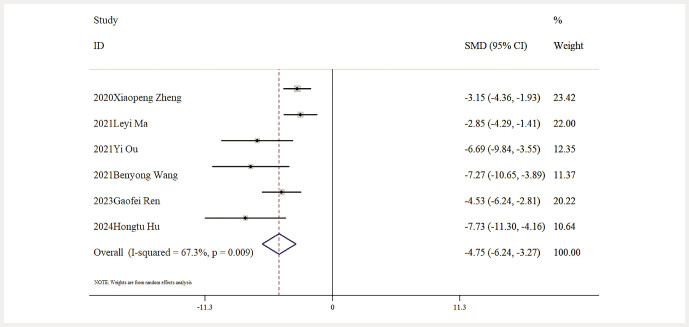
Forest plot: impact of baicalin on IL-6 level 6 , 13 , 14 , 17 , 19 , 22 .

**Fig. 11:**
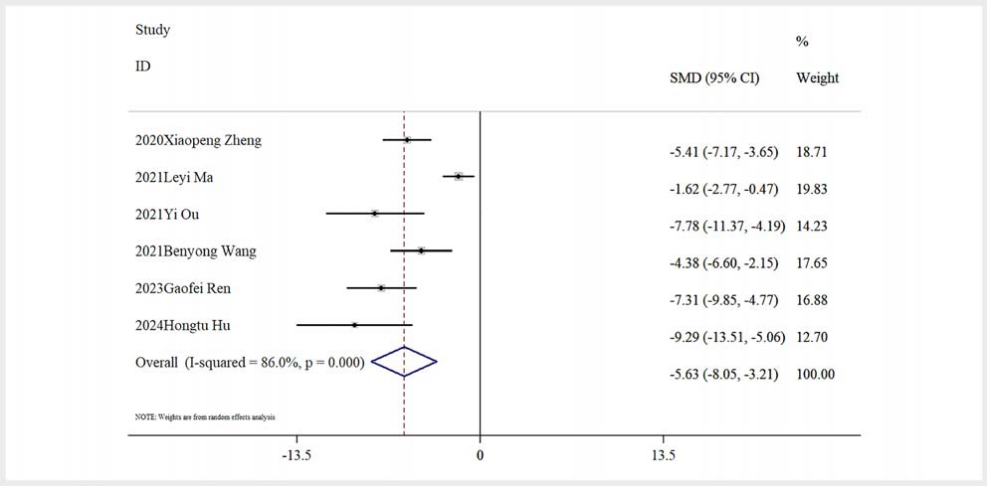
Forest plot: impact of baicalin on IL-1 *β* level 6 , 13 , 14 , 17 , 19 , 22 .

**Fig. 12:**
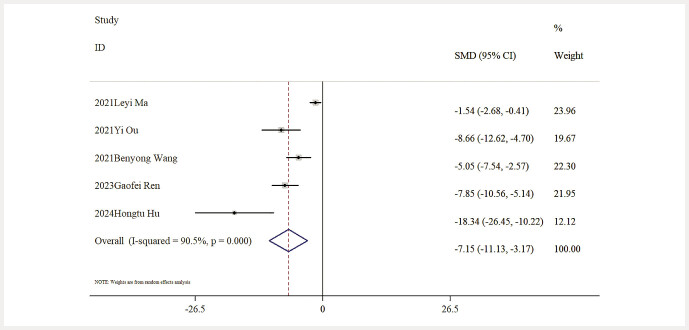
Forest plot: impact of baicalin on TNF- *α* level 6 , 13 , 14 , 19 , 22 .

Influence of Baicalin on Inflammatory Markers. Six studies provided data about IL-6 levels. The outcomes of a comprehensive analysis indicated that, relative to the model group, baicalin significantly lowered IL-6 levels [n = 97, SMD: − 4.75 (95% CI: − 6.24, − 3.27), P < 0.05; heterogeneity: I² = 67.3%, P = 0.009; Fig. 10 . Furthermore, data on IL-1 *β* levels were reported by six studies, and the analysis revealed a significant reduction in IL-1 *β* levels due to baicalin compared to the model group [n = 97, SMD: − 5.63 (95% CI: − 8.05, − 3.21), P < 0.05; heterogeneity: I² = 86%, P < 0.01; Fig. 11 . Additionally, five studies reported findings on TNF- *α* levels, with results demonstrating a significant decline in TNF- *α* levels for baicalin relative to the model group [n = 72, SMD: − 7.15 (95% CI: − 11.13, − 3.17), P < 0.05; heterogeneity: I² = 90.5%, P < 0.01; Fig. 12 .

**Fig. 13:**
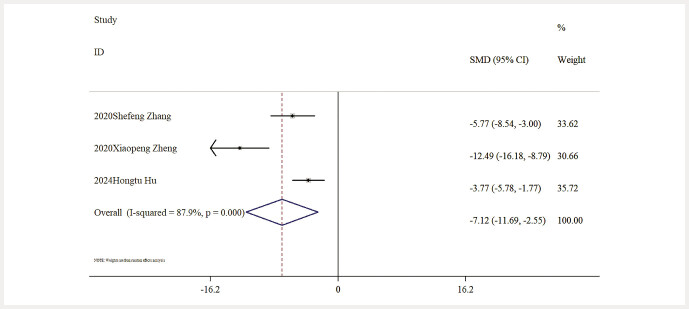
Forest plot: impact of baicalin on FN level 6 , 13 , 14 , 17 , 18 .

Influence of Baicalin on Renal Fibrosis. Data on FN levels were reported in three studies, showing that, when compared to the model group, baicalin significantly reduced FN levels [n = 49, SMD: − 7.12 (95% CI: − 11.69, − 2.55), P < 0.05; heterogeneity: I² = 87.9%, P < 0.01; Fig. 13 .

**Fig. 14:**
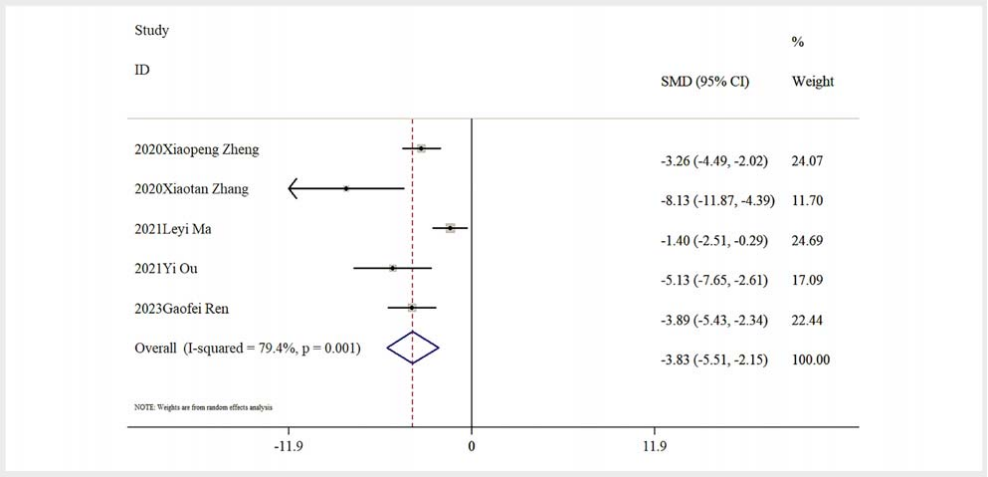
Forest plot: impact of baicalin on MDA level 6 , 12 , 13 , 14 , 18 .

**Fig. 15:**
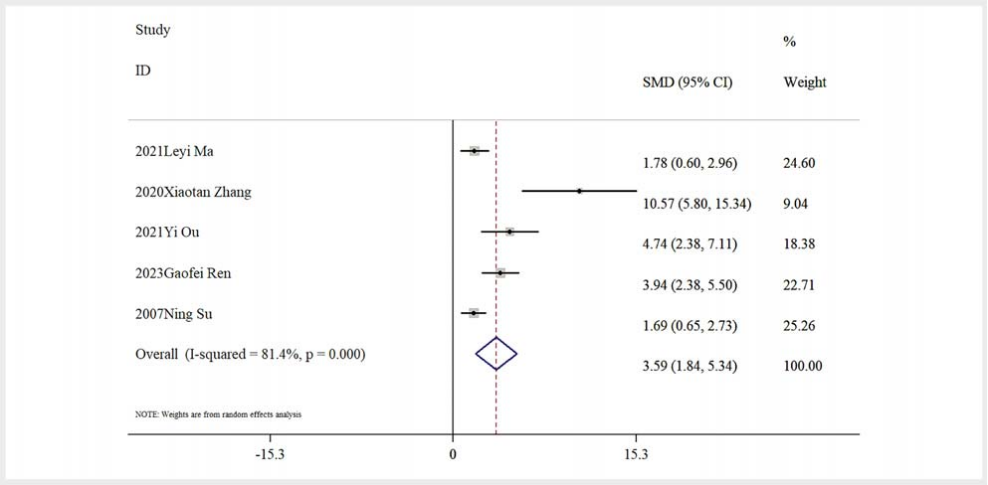
Forest plot: impact of baicalin on SOD level 17 , 19 , 21 .

Influence of Baicalin on Oxidative Stress. Five studies provided data on MDA levels, and the comprehensive analysis indicated that baicalin significantly lowered MDA levels compared to the model group [n = 85, SMD: − 3.83 (95% CI: − 5.51, − 2.15), P < 0.05; heterogeneity: I² = 79.4%, P < 0.01; Fig. 14 . Moreover, five studies reported data concerning SOD activity levels, with results indicating that baicalin significantly enhanced SOD activity when compared to the model group [n = 80, SMD: 3.59 (95% CI: 1.84, 5.34), P < 0.05; heterogeneity: I² = 81.4%, P < 0.01; Fig. 15 .

### Sensitivity analysis

A sensitivity analysis was conducted on key outcome measures, namely BUN, SCR, and FBG, to assess the influence of each individual study on the overall effect size. By systematically excluding each study one at a time, it was determined that the overall effect sizes for BUN, SCR, and FBG demonstrated considerable stability, suggesting that the removal or inclusion of particular studies did not substantially influence the outcome assessment (Supplementary **Figure 2 – 4** ).

### Subgroup analysis

Given the considerable heterogeneity identified in the primary outcomes, we investigated the potential contributors to this variability by examining the relationships between BUN, SCR, and FBG with variables such as species of animals involved, modeling methodologies, duration of intervention, and dosage regimens of baicalin. The results indicated that the length of the intervention might be a contributing factor to the heterogeneity observed in BUN, whereas differences in animal species could account for the heterogeneity noted in SCR. Furthermore, modeling methodologies were found to potentially influence the variability in FBG (Supplementary **Figure 5 – 16** ).

### Publication bias

**Fig. 16:**
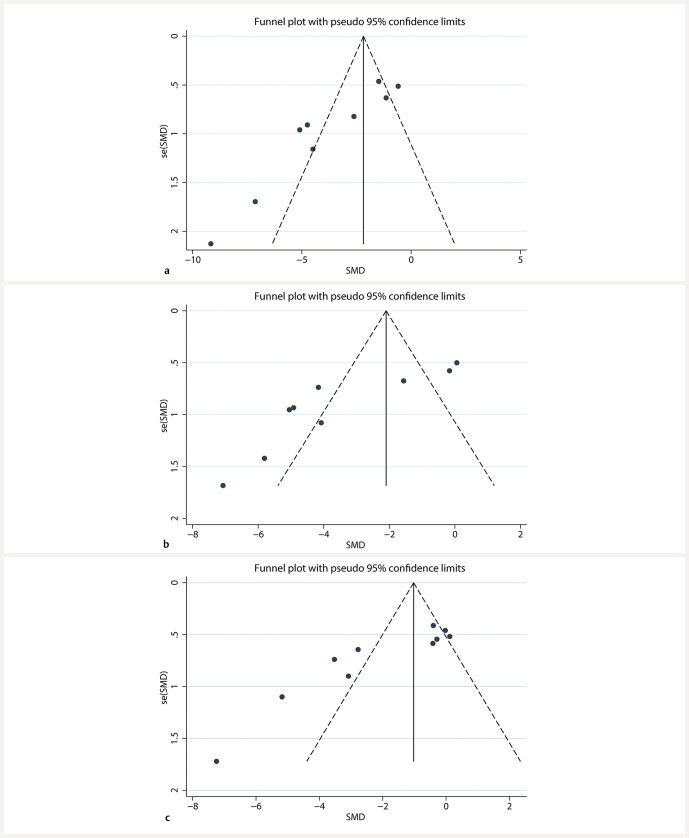
Funnel plot for ( **a** ) BUN, ( **b** ) SCR, ( **c** ) FBG.

**Fig. 17:**
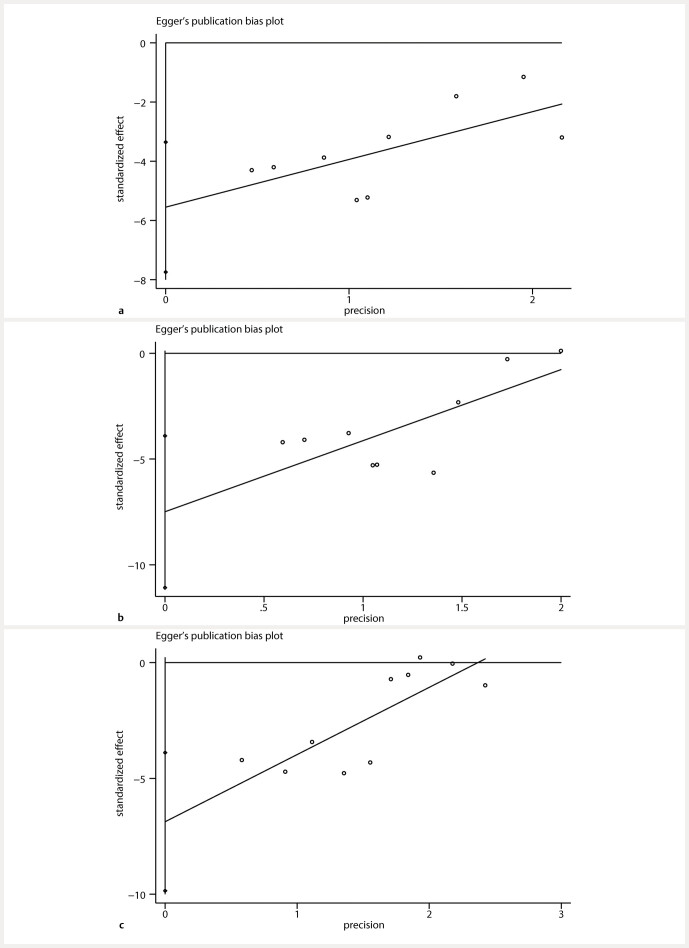
Eggerʼs publication bias plot for ( **a** ) BUN, ( **b** ) SCR, ( **c** ) FBG.

**Fig. 18:**
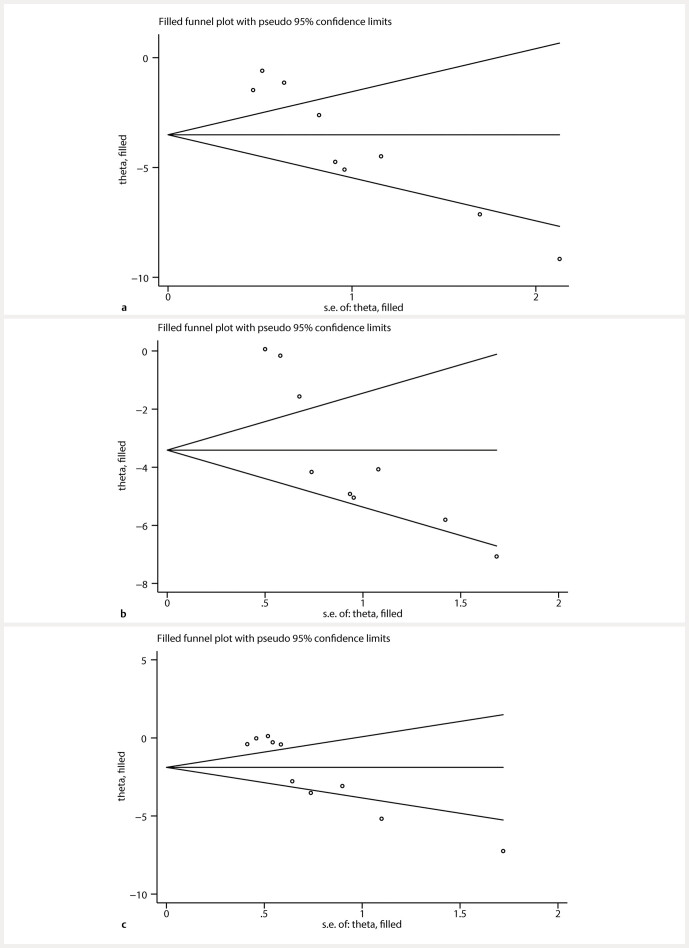
Trim-and-fill analysis for ( **a** ) BUN, ( **b** ) SCR, ( **c** ) FBG.

An assessment of publication bias was carried out concerning the primary outcome measures of BUN, SCR, and FBG. A visual examination of the funnel plot revealed asymmetry ( Fig. 16 ). Additionally, results from the Egger test ( Fig. 17 ) indicated a statistically significant presence of publication bias for BUN, SCR, and FBG (P < 0.05). Following the implementation of the trim-and-fill method for data adjustment, findings illustrated that the significance of the effect sizes remained consistent both before and after the trimming, thereby indicating stability ( Fig. 18 ). This observation implies that publication bias has not notably affected the results of this meta-analysis.

### Dose–time–response visualization

**Fig. 19:**
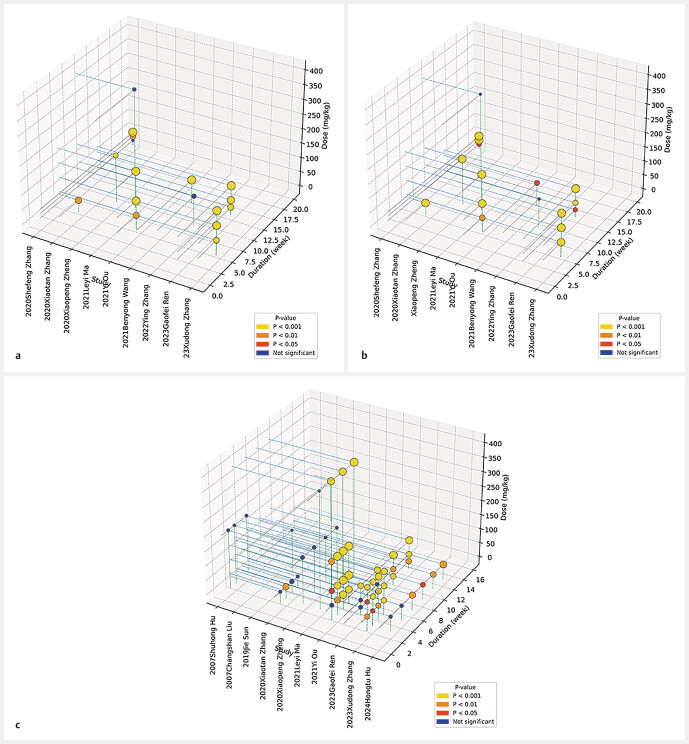
Dose–time–response plot of baicalin on ( **a** ) BUN, ( **b** ) SCR, ( **c** ) FBG. Each bubble represents a treatment subgroup with statistically significant improvement (P < 0.05). Bubble size corresponds to effect magnitude; x-axis indicates dose (mg/kg), and y-axis represents intervention duration (weeks).

To further evaluate the pharmacodynamic characteristics of baicalin, a dose–time–response analysis was performed for key outcome measures, including BUN, SCR, and FBG. Statistical significance was defined as P < 0.05. For FBG, baicalin exhibited significant glucose-lowering effects across a dose range of 25 to 400 mg/kg, with effective treatment durations between 1 and 12 weeks. Significant reductions in BUN were observed at doses of 25 to 200 mg/kg over intervention periods ranging from 4 to 20 weeks. For SCR, baicalin also showed significant effects at doses between 15 and 200 mg/kg, with treatment durations of 4 to 20 weeks. Taken together, these findings indicate that baicalin exerts a distinct dose- and time-dependent therapeutic effect on glycemic control and renal functional parameters in diabetic nephropathy models ( Fig. 19 ).

## Discussion

### Summary of evidence

We conducted a systematic meta-analysis of 14 preclinical studies (involving a total of 221 animals), and the results demonstrated that baicalin exerted a protective effect on DN animal models. By aggregating and analyzing the outcome data, we found that baicalin improved DN-related biochemical parameters, such as the renal function indicators BUN and SCR, UP, FBG, and lipid metabolism levels, including TC and TG. Furthermore, baicalin also improved certain mechanism-related indicators, such as inflammatory markers (IL-6, IL-1 *β* , and TNF- *α* ), oxidative stress markers (SOD and MDA), and fibrosis indicators (FN). These findings suggest that the protective mechanisms of baicalin in DN are likely associated with anti-inflammatory, antioxidant, and anti-fibrotic effects. However, our meta-analysis revealed high heterogeneity among the primary outcome measures, including BUN, SCR, and FBG. Therefore, we performed subgroup analyses, which indicated that the heterogeneity might be related to modeling methods, animal species, and duration of intervention. Additionally, considering the publication bias associated with BUN, SCR, and FBG, we employed the trim-and-fill method to supplement potential missed studies. The adjusted results did not show significant changes, indicating the stability of the findings.

### Mechanistic analysis

Based on the systematic analysis of the included literature, the potential mechanisms by which baicalin exerts a protective effect on DN animal models are primarily identified as follows.

### Anti-inflammatory effect

The experimental investigationʼs results suggest that signaling molecules linked to inflammatory processes and the release of pro-inflammatory factors are integral to the initiation and development of DN 23 . Inflammasomes, which are assemblies of several proteins situated within the cytoplasm, play a pivotal role in regulating the host immune response to harmful pathogens and molecules associated with cellular damage. Notably, within this group, the NOD-like receptor family pyrin domain containing 3 (NLRP3) inflammasome is uniquely activated by a range of inflammatory stimuli 24 . Multiple studies have demonstrated that baicalin can attenuate the activation of the NLRP3 inflammasome 25 , 26 , thereby disrupting its assembly and the subsequent maturation of pro-inflammatory cytokines.

**Fig. 20:**
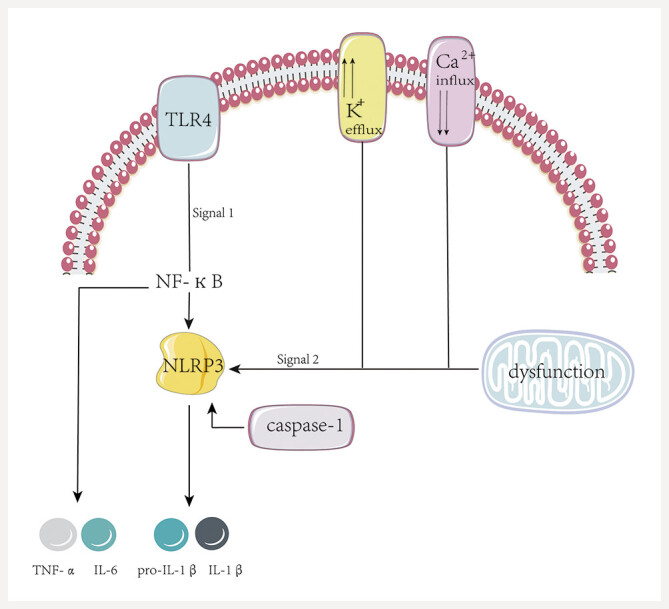
Inflammatory Pathway Diagram. TLR4, Toll-like receptor 4; NLRP3, NOD-like receptor family pyrin domain containing 3; caspase-1, cysteine-aspartic acid protease 1; TNF- *α* , tumor necrosis factor-alpha; IL-6, Interleukin-6; IL-1 *β* , Interleukin-1 beta; Pro-IL-1 *β* , Pro-interleukin-1 beta; NF- *κ* B, Nuclear Factor kappa-light-chain-enhancer of activated B cells.

The NLRP3 activation process necessitates two types of signals: the initial triggering signal (signal 1) and the subsequent activation signal (signal 2) ( Fig. 20 ). The initiation phase is chiefly mediated by Toll-like receptors (TLRs), a group of single-pass transmembrane proteins distinguished by their structure, which includes extracellular, transmembrane, and intracellular domains. Upon binding to their respective ligands, TLRs undergo dimerization or conformational changes that activate TLR signaling pathways, initiating the recruitment of downstream signaling molecules and ultimately leading to the activation of NF- *κ* B, which subsequently promotes NLRP3 expression 27 , 28 . Studies have shown that baicalin can inhibit the TLR4/NF- *κ* B signaling axis by suppressing TLR4 expression and preventing NF- *κ* B p65 nuclear translocation, thereby reducing NLRP3 priming and the transcription of pro-inflammatory cytokines 5 , 13 , 29 .

Pathogen-associated molecular patterns (PAMPs) 30 , 31 , 32 , 33 and danger-associated molecular patterns (DAMPs) 28 , 34 , 35 , 36 , 37 , 38 trigger activation signals, resulting in cellular disturbances such as ion flux alterations, mitochondrial dysfunction, or phagolysosomal rupture. NLRP3 functions as a sensor for these molecular patterns. Detection of these physiological changes by NLRP3 is facilitated through the involvement of NEK7 kinase, ASC, and caspase-1, leading to the formation of the NLRP3 inflammasome 39 , 40 , 41 . Caspase-1 processes gasdermin D, thereby releasing fragments that create pores in cell membranes and initiate pyroptosis. Additionally, active caspase-1 can cleave precursors of pro-inflammatory cytokines, notably pro-IL-1 *β* and IL-1 *β* , allowing their release through gasdermin D-created pores, which amplifies the inflammatory response 42 , 43 . Baicalin treatment has been reported to reduce caspase-1 cleavage and IL-1 *β* maturation, thereby inhibiting pyroptosis and inflammation in renal tubular epithelial cells 44 . In addition, baicalin can preserve mitochondrial function and suppress the production of reactive oxygen species (ROS) 45 , 46 , both of which are critical upstream events in DAMP-induced NLRP3 activation.

Furthermore, the activation of NF- *κ* B results in the production of various pro-inflammatory cytokines, such as TNF- *α* and IL-6, which not only promote inflammation 47 , 48 but also recruit immune cells to eradicate pathogens or heal damaged tissue. Multiple experimental studies have demonstrated that baicalin effectively suppresses the renal expression of TNF- *α* and IL-6 in diabetic nephropathy models 13 , 14 , 15 , 19 , 22 , thereby mitigating immune cell infiltration and preserving renal structural integrity.

### Anti-fibrosis

**Fig. 21:**
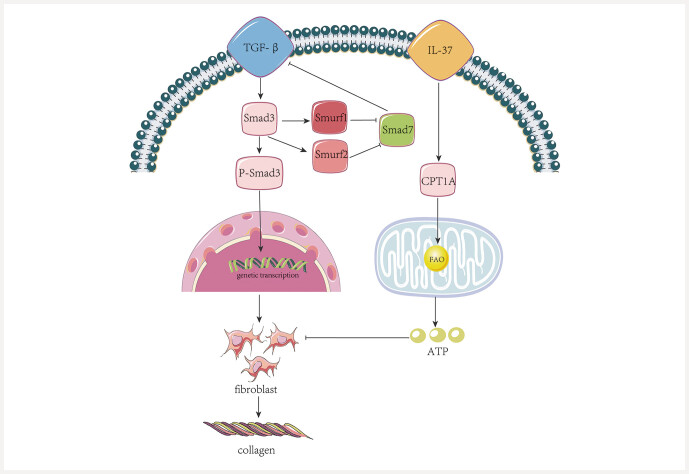
Fibrosis Mechanism Diagram. TGF- *β* , Transforming Growth Factor Beta; Smad3, Mothers Against Decapentaplegic Homolog 3; Smurf1, SMAD Ubiquitination Regulatory Factor 1; Smurf2, SMAD Ubiquitination Regulatory Factor 2; Smad7, SMAD Family Member 7; IL-37, Interleukin-37; CPT1A, Carnitine Palmitoyltransferase 1A; FAO, Fatty Acid Oxidation.

Renal fibrosis refers to the gradual build-up of scar tissue within the renal parenchyma, caused by the unrestrained deposition of fibrotic matrix components during chronic injury periods. This pathological condition impairs the structural integrity of the kidneys, reduces blood flow, compromises organ functionality, and can ultimately culminate in renal failure. It is recognized as a common final pathway for most chronic and progressive kidney diseases. Interstitial fibrosis is typified by the widening of space between the tubular basement membrane and peritubular capillaries, resulting from the excessive accumulation of extracellular matrix proteins. Collagen type I is the main matrix protein prevalent in renal fibrosis, although other collagen types such as III, V, VI, VII, and XV, along with the adhesive glycoprotein fibronectin, are also found in considerable quantities 49 . Activated fibroblasts are primarily responsible for producing these proteins 50 . Alpha-smooth muscle actin ( *α* -SMA) serves as a crucial marker for myofibroblasts, reflecting their increased responsiveness to transforming growth factor-beta 1 (TGF- *β* 1), a key regulatory element in myofibroblast differentiation during fibrosis. TGF- *β* 1 is considered a fundamental driver that initiates renal fibrosis by activating Smad proteins, notably Smad3. When activated by TGF- *β* 1 or other stressors, Smad3 translocates to the nucleus where it binds to specific DNA regions to regulate the expression of target genes involved in fibrogenesis ( Fig. 21 ), such as those associated with collagen synthesis and epithelial-mesenchymal transition (EMT) 51 , 52 . Previous studies have confirmed that baicalin can reverse epithelial-to-mesenchymal transition (EMT) by inhibiting the TGF- *β* /Smad3 signaling pathway 53 , 54 .

Moreover, Smad3 influences Smad7 within the context of renal fibrosis. Smad7, an inhibitory Smad, is transcriptionally activated by Smad3 and plays a role in moderating the TGF- *β* /Smad signaling via negative feedback 55 , 56 , 57 , 58 . Under normal physiological conditions, Smad7 is abundantly present in the kidneys, facilitating the degradation of type I TGF- *β* receptors through the ubiquitin-proteasome pathway, thus exerting its inhibitory effects and preventing Smad3 recruitment and phosphorylation. In pathological scenarios, hyperactivated Smad3 can induce various E3 ubiquitin ligases, such as Smad ubiquitination regulatory factor 1 (Smurf1) and factor 2 (Smurf2). These ligases interact with Smad7, promoting its degradation via a ubiquitin-dependent mechanism 59 , 60 . This degradation results in elevated TGF- *β* /Smad3 signaling, which contributes to the advancement of renal fibrosis ( Fig. 21 ). In recent years, both *in vivo* and *in vitro* experiments have confirmed that baicalin can attenuate renal interstitial fibrosis in mice by inhibiting the TGF- *β* /Smad3 signaling pathway 61 , 62 . This is achieved by suppressing Smad3 phosphorylation and nuclear translocation in fibroblasts, thereby downregulating the expression of fibrosis-related genes.

Additionally, renal tubular epithelial cells (TECs) are the most metabolically active cells within the renal parenchyma, playing a vital role in the active reabsorption of significant amounts of solutes, which necessitates high energy levels 63 . Fatty acid oxidation (FAO) serves as the fundamental energy source for TECs, and any deficiency in FAO results in ATP depletion, lipid buildup, and immune cell infiltration, eventually leading to renal fibrosis 64 . Studies have indicated that baicalin can alleviate lipid accumulation by upregulating FAO activity and enhancing ATP production 65 . Carnitine palmitoyltransferase 1A (CPT1A) acts as a key enzyme in this pathway. Intracellular IL-37 can enhance the expression of CPT1A, thus facilitating FAO restoration, boosting ATP levels, and decreasing lipid droplet accumulation, which collectively diminishes the expression of fibrosis markers and restrains renal fibrosis in DN 66 . Proteomics studies have demonstrated that baicalin accelerates mitochondrial lipid oxidation by targeting CPT1A, thereby reducing lipid deposition 67 . In addition, evidence suggests that baicalin may function as a CPT1A activator to enhance FAO, support energy production, and mitigate renal fibrosis 68 .

## Antioxidant

The kidneys, owing to their substantial oxygen usage, are particularly susceptible to oxidative-stress-related damage. This condition emerges from an imbalance between oxidants and antioxidants, which results in an excessive accumulation of reactive oxygen species (ROS). Although moderate elevations in ROS levels can be managed, exceeding the bodyʼs intrinsic regulatory abilities might lead to cellular damage or apoptosis 69 . ROS encompasses various types, including superoxide anions, hydrogen peroxide (H2O2), nitric oxide (NO), hydroxyl radicals, and peroxynitrite 70 . The mitochondrial respiratory chain is the primary driver of ROS production, positioning mitochondria as a major source of these reactive entities 71 . Baseline levels of ROS are vital for numerous cellular and tissue functions, such as gene expression, molecular transcription, and signal transduction 72 . However, the overproduction of ROS can hasten the progression of pathological conditions like DN. Antioxidant defense mechanisms can be initiated to mitigate ROS emanating from diverse sources. Central antioxidant enzymes include superoxide dismutase (SOD), glutathione peroxidase (GSH-Px), catalase (CAT), glutathione reductase (GR), and peroxiredoxins 73 . Indicators of oxidative harm, such as malondialdehyde (MDA) and protein carbonyls, can worsen oxidative injury within the organism.

**Fig. 22:**
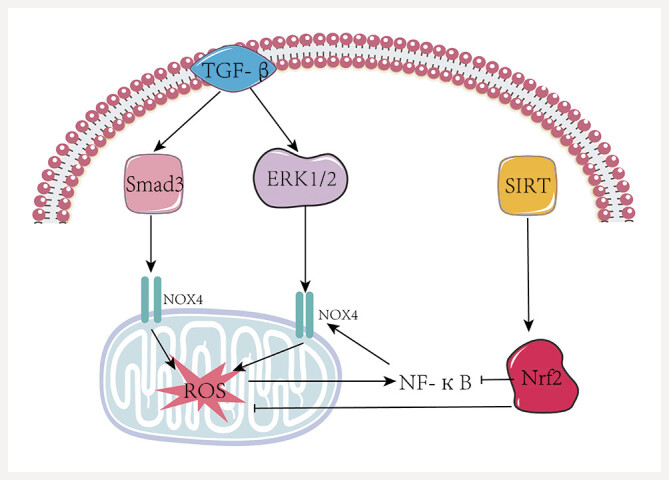
Oxidative Stress Mechanism Diagram. TGF- *β* , Transforming Growth Factor Beta; NF- *κ* B, Nuclear Factor kappa-light-chain-enhancer of activated B cells; Smad3, Mothers Against Decapentaplegic Homolog 3; EPK1/2, Extracellular Signal-Regulated Kinase 1/2; NOX4, NADPH oxidase 4; SIRT, Silent Information Regulator; Nrf2, Nuclear Factor Erythroid 2-Related Factor 2.

Oxidative stress significantly contributes to the etiology, pathogenesis, and progression of DN. Hyperglycemia stimulates the polyol pathway and the formation of advanced glycation end products (AGEs), alongside their receptor (RAGE) and protein kinase C (PKC) involvement. The AGEs-RAGE signaling pathway activation increases NADPH oxidase (NOX) activity, thus amplifying ROS production and exacerbating oxidative stress 74 . NADPH oxidases (NOXs), particularly Nox4, are primary enzymatic sources of ROS in kidney tissues. Nox4 can be upregulated in renal tissues via the TGF- *β* signaling pathway, where TGF- *β* -induced Smad3 and extracellular signal-regulated kinases (ERK1/2)–members of the MAPK family–activation results in increased Nox4 expression 75 , 76 ( Fig. 22 ). Studies have shown that baicalin treatment can modulate NOX4 expression in endothelial injury models, thereby reducing the production of reactive oxygen species (ROS) 77 . Moreover, baicalin has been found to attenuate ROS levels and alleviate pathological damage through inhibition of the TGF- *β* /Smad3 78 and ERK 79 signaling pathways. This upregulation of Nox4 causes excessive ROS accumulation and triggers downstream signaling cascades, including ERK, mTOR, PKB, and MAPK, promoting inflammation and kidney fibrosis. Elevated ROS levels can activate the NF- *κ* B pathway, creating a feedback loop where NF- *κ* B activation further enhances NOX expression and thereby increases ROS production 80 . For instance, evidence suggests that baicalin may reduce ROS generation by enhancing the production of antioxidant enzymes through modulation of the TGF- *β* 1/NOX4/NF- *κ* B signaling axis 81 .

Oxidative stress involves not only ROS accumulation but also a reduction in antioxidant defenses. As a key redox-sensitive transcription factor, nuclear factor erythroid 2-related factor 2 (Nrf2) boosts the expression of antioxidant proteins like glutathione S-transferases, heme oxygenase-1, catalase, gamma-glutamyl cysteine synthetase, and NAD(P)H quinone oxidoreductase, thus mitigating ROS levels 82 . Previous studies have demonstrated that baicalin can significantly upregulate antioxidant enzyme expression via the Nrf2 signaling pathway, thereby alleviating oxidative stress in diabetic nephropathy 6 . The protective effects of Nrf2 on the kidneys have been demonstrated in several animal models of chronic kidney disease 83 , 84 , 85 . Nrf2 not only positively regulates antioxidant proteins but also inhibits NF- *κ* B activity, with this modulation being mediated by SIRT signaling pathways 86 . Evidence has further indicated that baicalin exerts antioxidant effects through modulation of the SIRT signaling pathway 87 , 88 .

## Limitations and Future Perspectives

This study evaluated the therapeutic efficacy of baicalin in DN based on preclinical evidence, thereby laying a foundation for its potential clinical application. However, several limitations should be acknowledged. First, although animal models can provide valuable mechanistic insights, differences in species, strains, modeling methods, dosing protocols, and experimental conditions may introduce substantial heterogeneity. Such variability could affect the overall effect size and reduce the generalizability of the conclusions. Second, the number of included studies and total sample size were relatively limited, with only 14 studies and 221 animals meeting the inclusion criteria. This limited evidence base may compromise the statistical power of the meta-analysis and weaken the robustness of the conclusions. In preclinical research, small sample sizes also increase the risk of publication bias and may lead to an overestimation of therapeutic efficacy. Third, although rigorous efforts were made to extract data accurately, some results were derived using the WebPlotDigitizer tool. Minor deviations may still exist, even after independent extraction and cross-verification by two researchers. Moreover, several studies lacked sufficient details regarding randomization, blinding, and allocation concealment, which introduces uncertainty in the risk of bias assessment. Finally, inherent differences between animal models and human DN–such as variations in pathological mechanisms, metabolic profiles, immune responses, and pharmacokinetics–limit the direct extrapolation of findings to clinical contexts and must be interpreted with caution.

Future studies should involve larger sample sizes, improved methodological rigor, and validation across multiple animal species. In addition, integrating multi-omics approaches may offer deeper insights into the pharmacological mechanisms of baicalin and accelerate its clinical translation.

## Contributorsʼ Statement

Data collection: An, Hengtong, Liu, Luyao,He, Tongtong, Chen, Xiaohan, Jin, Xiaofei design of the study: An, Hengtong, Liu, Luyao, He, Tongtong, Chen, Xiaohan, Zhou, Xiaohong, Gao, Weijuan statistical analysis: An, Hengtong, Liu, Luyao, He, Tongtong, Chen, Xiaohan, Jin, Xiaofei, Zhou, Xiaohong, Gao, Weijuan analysis and interpretation of the data: An, Hengtong, Liu, Luyao,He, Tongtong, Chen, Xiaohan, Jin, Xiaofei, Zhou, Xiaohong, Gao, Weijuan drafting the manuscript: An, Hengtong critical revision of the manuscript: Jin, Xiaofei, Zhou, Xiaohong, Gao, Weijuan

## Supporting Information

Ergänzendes Material Supporting Information
