# Supplementary material for: Preclinical Evaluation of Baicalin for the Treatment of Diabetic Nephropathy: A Systematic Review and Meta-Analysis
Source: Planta Med. 2025 Jul 24;91(13):706–29. doi: 10.1055/a-2615-8249 (PMC12527465; doi:10.1055/a-2615-8249)
Supplement: Supplementary file 1 — Ergänzendes Material [file 10-1055-a-2615-8249-sup_pmj0139.pdf]

supplementary materials

search strategy

PubMed:

("diabetic nephropathies"[MeSH Terms] OR ("diabetic"[All Fields] AND "nephropathies"[All Fields]) OR "diabetic nephropathies"[All Fields] OR ("diabetic nephropathies"[MeSH Terms] OR ("diabetic"[All Fields] AND "nephropathies"[All Fields]) OR "diabetic nephropathies"[All Fields] OR ("nephropathies"[All Fields] AND "diabetic"[All Fields]) OR "nephropathies diabetic"[All Fields]) OR ("diabetic nephropathies"[MeSH Terms] OR ("diabetic"[All Fields] AND "nephropathies"[All Fields]) OR "diabetic nephropathies"[All Fields] OR ("nephropathy"[All Fields] AND "diabetic"[All Fields]) OR "nephropathy diabetic"[All Fields]) OR ("diabetic nephropathies"[MeSH Terms] OR ("diabetic"[All Fields] AND "nephropathies"[All Fields]) OR "diabetic nephropathies"[All Fields] OR ("diabetic"[All Fields] AND "kidney"[All Fields] AND "disease"[All Fields]) OR "diabetic kidney disease"[All Fields]) OR ("diabetic nephropathies"[MeSH Terms] OR ("diabetic"[All Fields] AND "nephropathies"[All Fields]) OR "diabetic nephropathies"[All Fields] OR ("diabetic"[All Fields] AND "kidney"[All Fields] AND "diseases"[All Fields]) OR "diabetic kidney diseases"[All Fields]) OR ("diabetic nephropathies"[MeSH Terms] OR ("diabetic"[All Fields] AND "nephropathies"[All Fields]) OR "diabetic nephropathies"[All Fields] OR ("kidney"[All Fields] AND "disease"[All Fields] AND "diabetic"[All Fields]) OR "kidney disease diabetic"[All Fields]) OR ("diabetic nephropathies"[MeSH Terms] OR ("diabetic"[All Fields] AND "nephropathies"[All Fields]) OR "diabetic nephropathies"[All Fields] OR ("kidney"[All Fields] AND "diseases"[All Fields] AND "diabetic"[All Fields]) OR "kidney diseases diabetic"[All Fields]) OR ("diabetic nephropathies"[MeSH Terms] OR ("diabetic"[All Fields] AND "nephropathies"[All Fields]) OR "diabetic nephropathies"[All Fields] OR ("diabetic"[All Fields] AND "nephropathy"[All Fields]) OR "diabetic nephropathy"[All Fields]) OR ("diabetic nephropathies"[MeSH Terms] OR ("diabetic"[All Fields] AND "nephropathies"[All Fields]) OR "diabetic nephropathies"[All Fields] OR ("diabetic"[All Fields] AND "glomerulosclerosis"[All Fields]) OR "diabetic glomerulosclerosis"[All Fields]) OR ("diabetic nephropathies"[MeSH Terms] OR ("diabetic"[All Fields] AND "nephropathies"[All Fields]) OR "diabetic nephropathies"[All Fields] OR ("glomerulosclerosis"[All Fields] AND "diabetic"[All Fields]) OR "glomerulosclerosis diabetic"[All Fields]) OR ("diabetic nephropathies"[MeSH Terms] OR ("diabetic"[All Fields] AND "nephropathies"[All Fields]) OR "diabetic nephropathies"[All

Planta Med | DOI 10.1055/a-2615-8249 | © The Author(s).

Fields] OR ("intracapillary"[All Fields] AND "glomerulosclerosis"[All Fields]) OR "intracapillary glomerulosclerosis"[All Fields]) OR ("diabetic nephropathies"[MeSH Terms] OR ("diabetic"[All Fields] AND "nephropathies"[All Fields]) OR "diabetic nephropathies"[All Fields] OR ("kimmelstiel"[All Fields] AND "wilson"[All Fields] AND "disease"[All Fields]) OR "kimmelstiel wilson disease"[All Fields]) OR ("diabetic nephropathies"[MeSH Terms] OR ("diabetic"[All Fields] AND "nephropathies"[All Fields]) OR "diabetic nephropathies"[All Fields] OR ("kimmelstiel"[All Fields] AND "wilson"[All Fields] AND "disease"[All Fields]) OR "kimmelstiel wilson disease"[All Fields]) OR ("diabetic nephropathies"[MeSH Terms] OR ("diabetic"[All Fields] AND "nephropathies"[All Fields]) OR "diabetic nephropathies"[All Fields] OR ("nodular"[All Fields] AND "glomerulosclerosis"[All Fields]) OR "nodular glomerulosclerosis"[All Fields]) OR ("diabetic nephropathies"[MeSH Terms] OR ("diabetic"[All Fields] AND "nephropathies"[All Fields]) OR "diabetic nephropathies"[All Fields] OR ("glomerulosclerosis"[All Fields] AND "nodular"[All Fields]) OR "glomerulosclerosis nodular"[All Fields]) OR ("diabetic nephropathies"[MeSH Terms] OR ("diabetic"[All Fields] AND "nephropathies"[All Fields]) OR "diabetic nephropathies"[All Fields] OR ("kimmelstiel"[All Fields] AND "wilson"[All Fields] AND "syndrome"[All Fields]) OR "kimmelstiel wilson syndrome"[All Fields]) OR ("diabetic nephropathies"[MeSH Terms] OR ("diabetic"[All Fields] AND "nephropathies"[All Fields]) OR "diabetic nephropathies"[All Fields] OR ("kimmelstiel"[All Fields] AND "wilson"[All Fields] AND "syndrome"[All Fields]) OR "kimmelstiel wilson syndrome"[All Fields]) OR (("syndrom"[All Fields] OR "syndromal"[All Fields] OR "syndromally"[All Fields] OR "syndrome"[MeSH Terms] OR "syndrome"[All Fields] OR "syndromes"[All Fields] OR "syndrome s"[All Fields] OR "syndromic"[All Fields] OR "syndroms"[All Fields]) AND "Kimmelstiel-Wilson"[All Fields])) AND ("baicalin"[Supplementary Concept] OR "baicalin"[All Fields] OR "baicalin s"[All Fields])

Web of Science：

Diabetic Nephropathies (Topic) or Nephropathies, Diabetic (Topic) or Nephropathy, Diabetic (Topic) or Diabetic Kidney Disease (Topic) or Diabetic Kidney Diseases (Topic) or Kidney Disease, Diabetic (Topic) or Kidney Diseases, Diabetic (Topic) or Diabetic Nephropathy (Topic) or Diabetic Glomerulosclerosis (Topic) or Glomerulosclerosis, Diabetic (Topic) or Intracapillary Glomerulosclerosis (Topic) or Kimmelstiel-Wilson Disease (Topic) or Kimmelstiel Wilson Disease (Topic) or Nodular Glomerulosclerosis (Topic) or Glomerulosclerosis, Nodular (Topic) or Kimmelstiel-Wilson Syndrome (Topic) or Kimmelstiel Wilson Syndrome (Topic) or Syndrome, Kimmelstiel-Wilson (Topic) and Preprint Citation Index (Exclude – Database)

Planta Med | DOI 10.1055/a-2615-8249 | © The Author(s).

AND

baicalin (Topic) and Preprint Citation Index (Exclude – Database)

Embase:

(Nephropathies, Diabetic or Nephropathy, Diabetic or Diabetic Kidney Disease or Diabetic Kidney Diseases or Kidney Disease, Diabetic or Kidney Diseases, Diabetic or Diabetic Nephropathy or Diabetic Glomerulosclerosis or Glomerulosclerosis, Diabetic or Intracapillary Glomerulosclerosis or Kimmelstiel-Wilson Disease or Kimmelstiel Wilson Disease or Nodular Glomerulosclerosis or Glomerulosclerosis, Nodular or Kimmelstiel-Wilson Syndrome or Kimmelstiel Wilson Syndrome or Syndrome, Kimmelstiel-Wilson).af.

AND

baicalin.af.

CNKI, Wangfang Data, VPCS:

Search terms included:“黄芩苷” (baicalin);“糖尿病肾病” (diabetic nephropathy)

Boolean operator AND was used to combine terms. No language restrictions were applied within Chinese databases.

supplementary Figure:

Planta Med | DOI 10.1055/a-2615-8249 | © The Author(s).

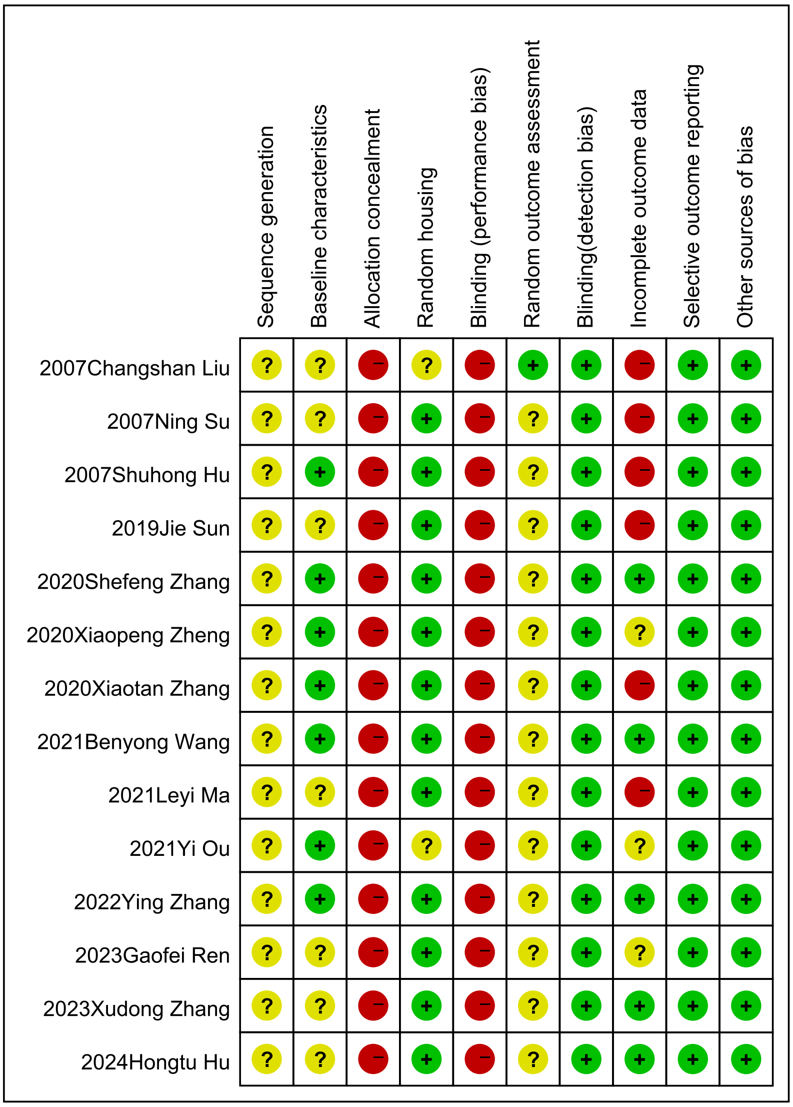

supplementary Figure 1. Risk of bias summary

Planta Med | DOI 10.1055/a-2615-8249 | © The Author(s).

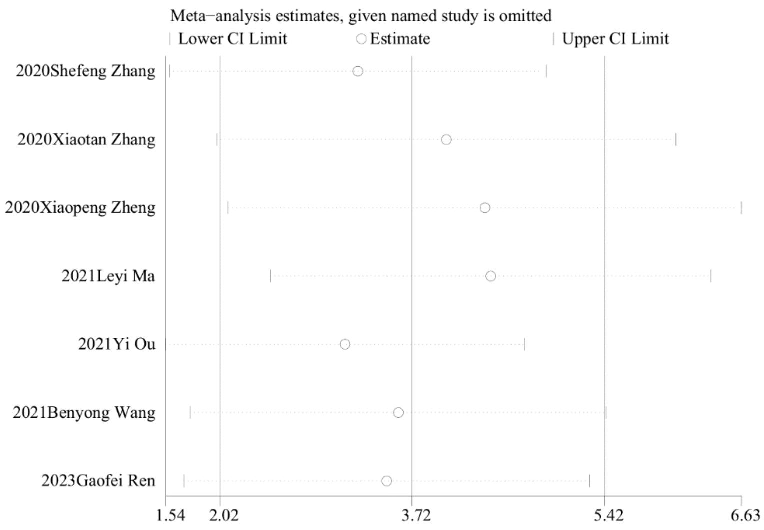

Supplementary Figure 2. sensitivity analysis of BUN

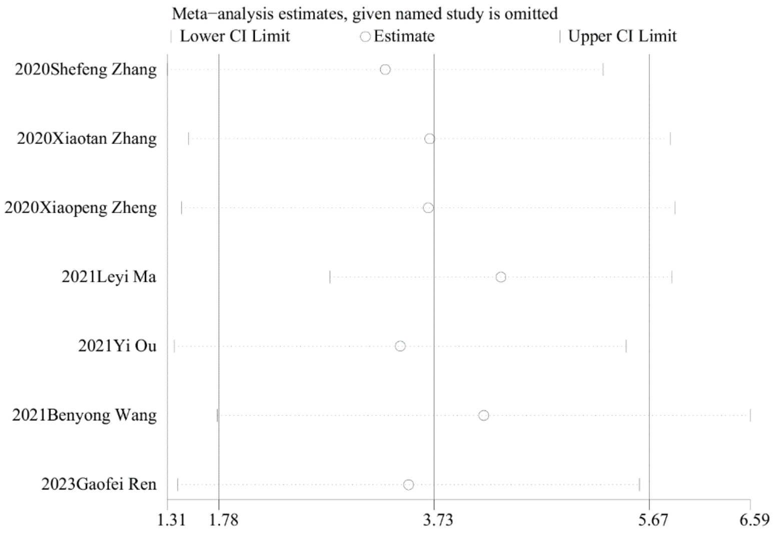

Supplementary Figure 3. sensitivity analysis of SCR

Planta Med | DOI 10.1055/a-2615-8249 | © The Author(s).

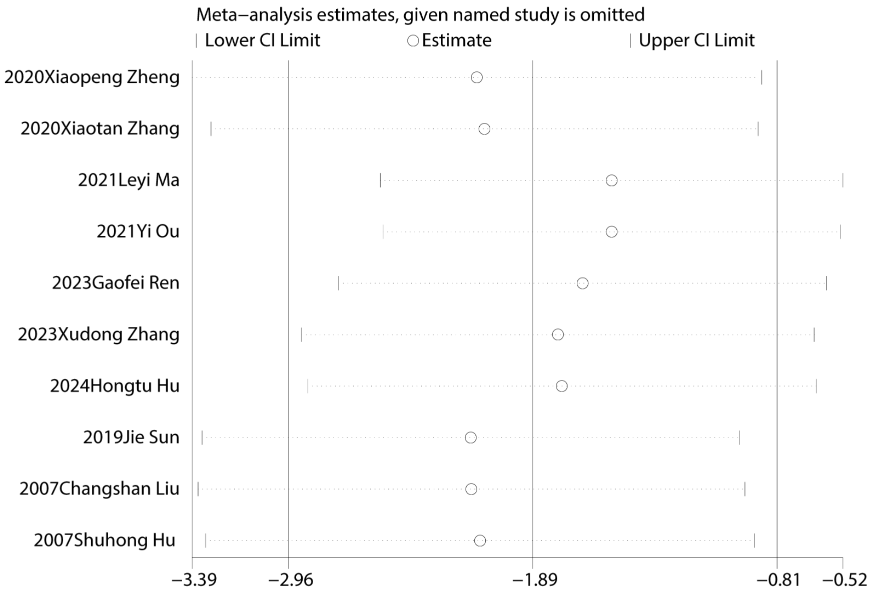

Supplementary Figure 4. sensitivity analysis of FBG

Planta Med | DOI 10.1055/a-2615-8249 | © The Author(s).

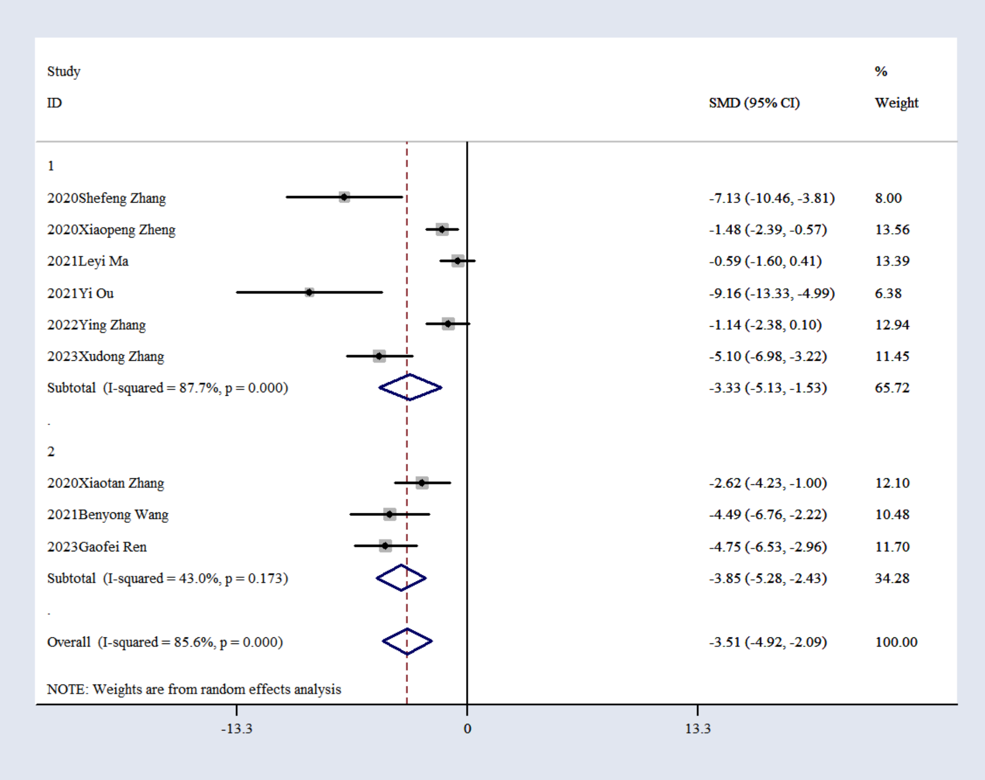

Supplementary Figure 5. Subgroup analysis of BUN based on intervention duration

Planta Med | DOI 10.1055/a-2615-8249 | © The Author(s).

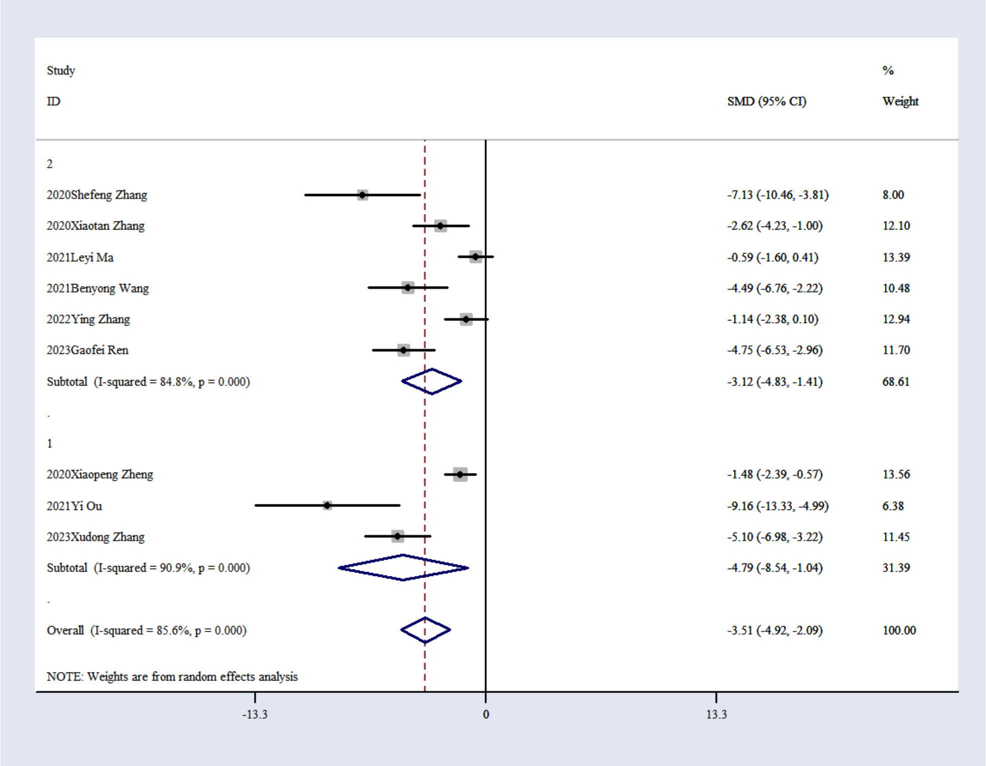

Supplementary Figure 6. Subgroup analysis of BUN based on species of animals

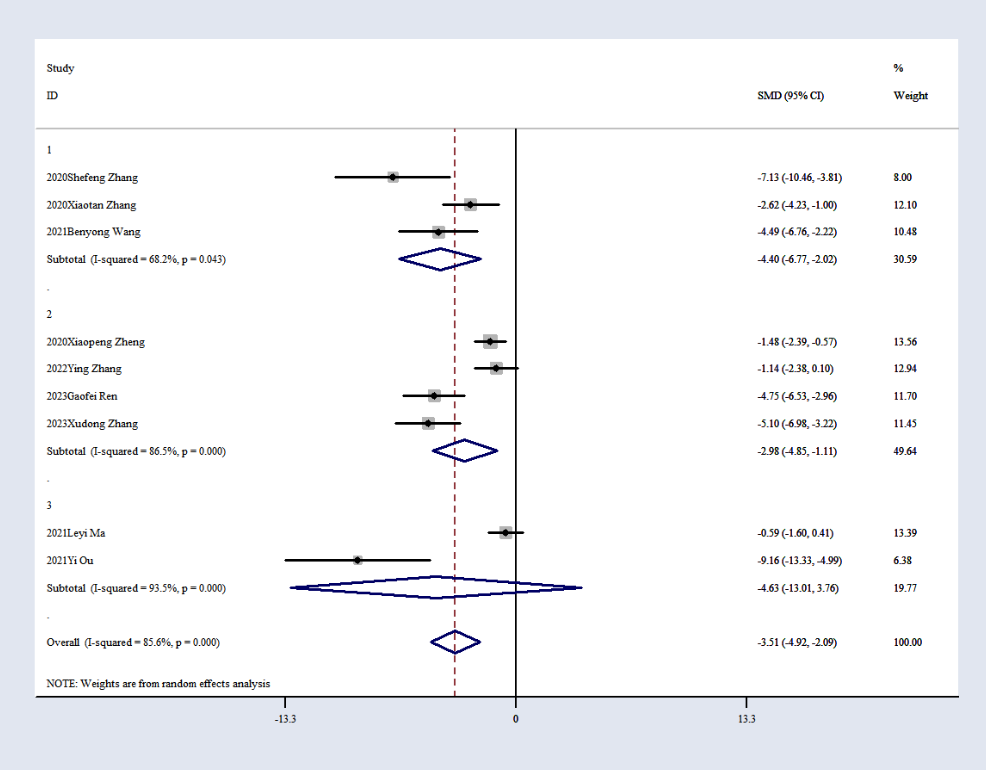

Planta Med | DOI 10.1055/a-2615-8249 | © The Author(s).

Supplementary Figure 7. Subgroup analysis of BUN based on dosage regimens of baicalin

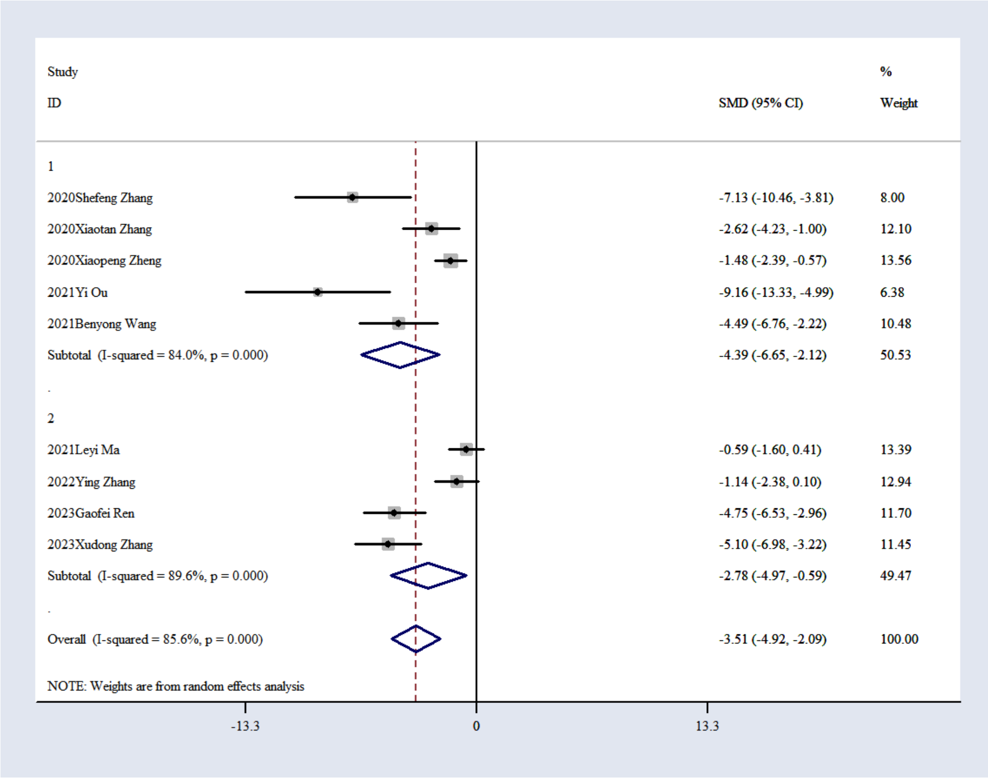

Supplementary Figure 8. Subgroup analysis of BUN based on modeling methodologies

Planta Med | DOI 10.1055/a-2615-8249 | © The Author(s).

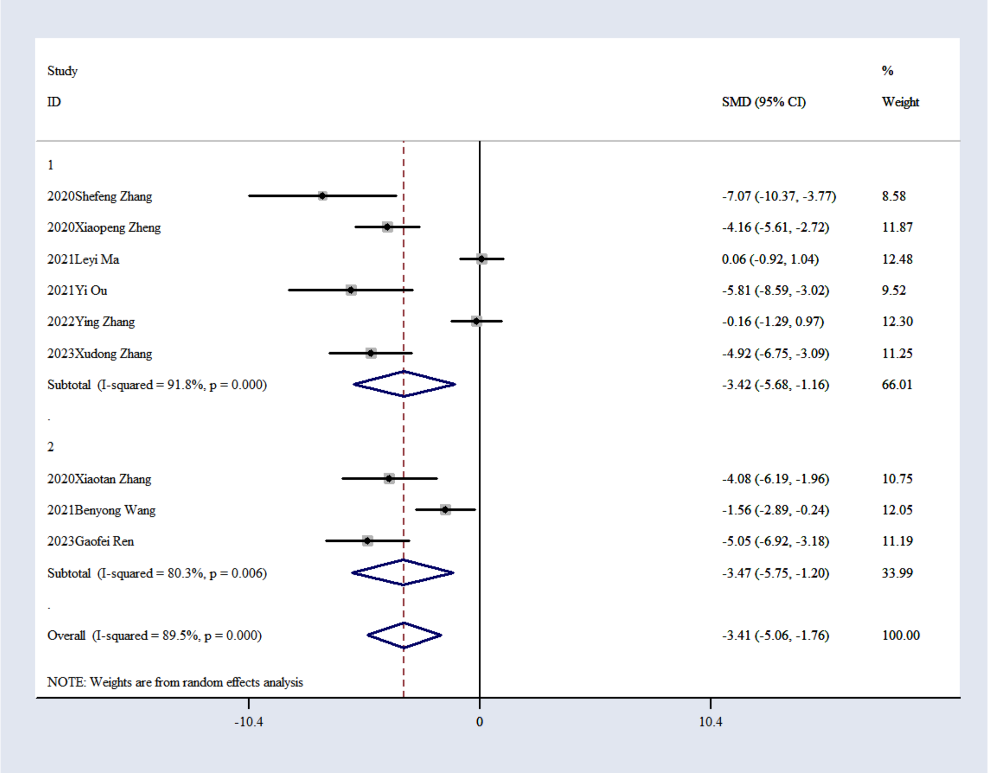

Supplementary Figure 9. Subgroup analysis of SCR based on intervention duration

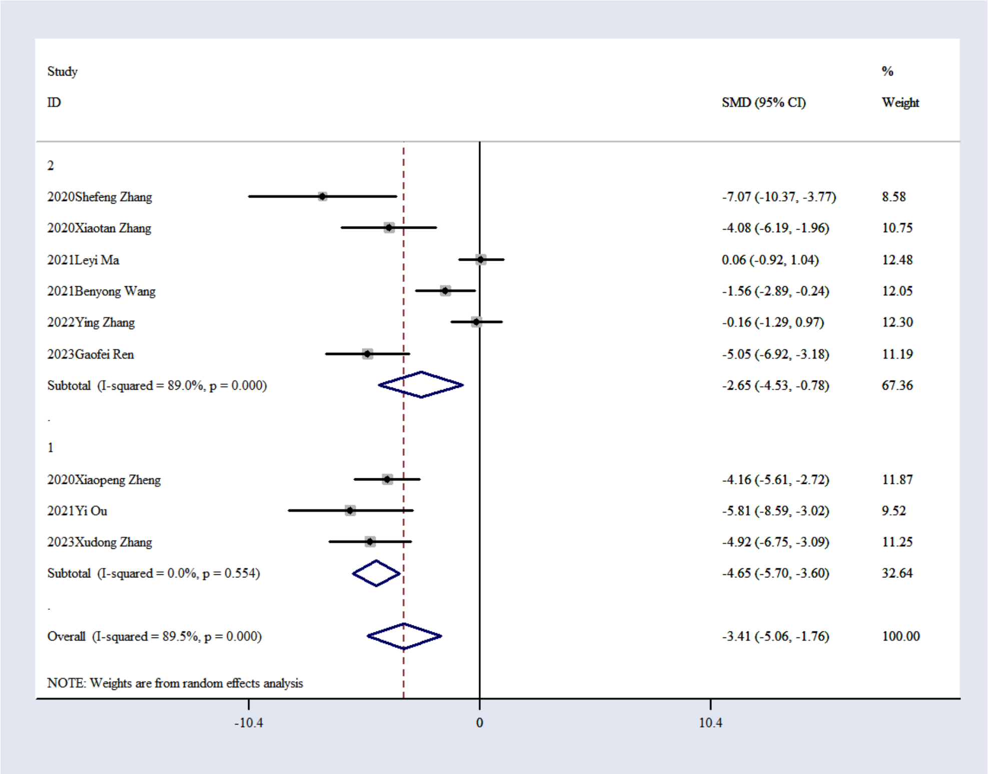

Planta Med | DOI 10.1055/a-2615-8249 | © The Author(s).

Supplementary Figure 10. Subgroup analysis of SCR based on species of animals

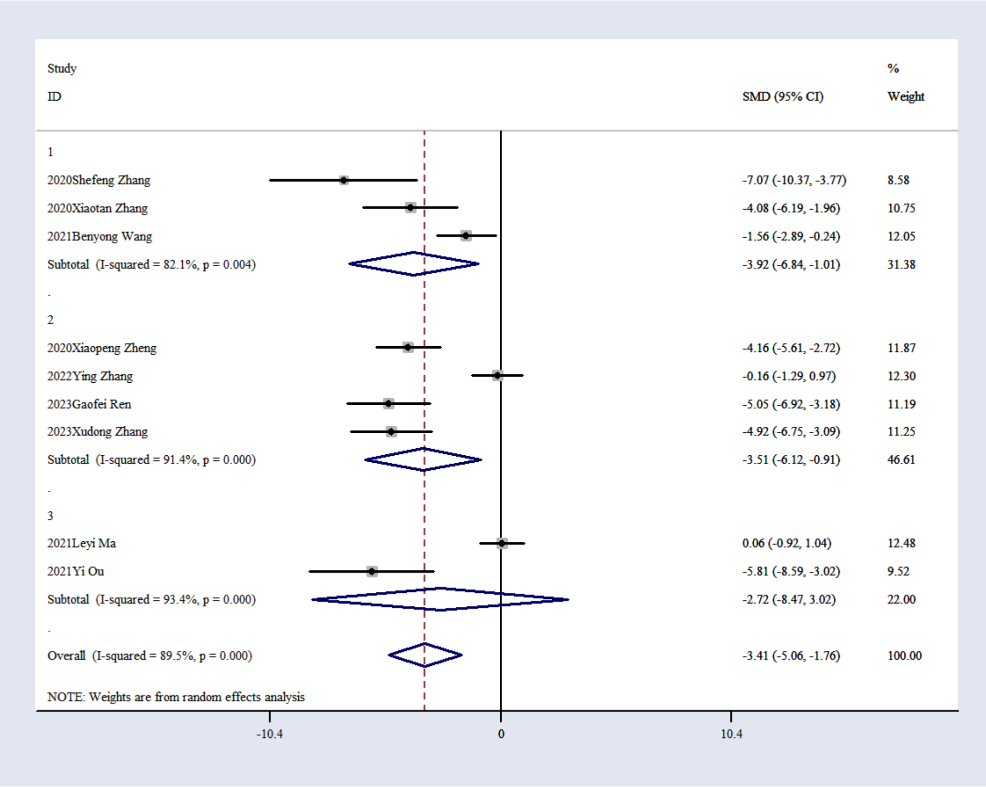

Supplementary Figure 11. Subgroup analysis of SCR based on dosage regimens of baicalin

Planta Med | DOI 10.1055/a-2615-8249 | © The Author(s).

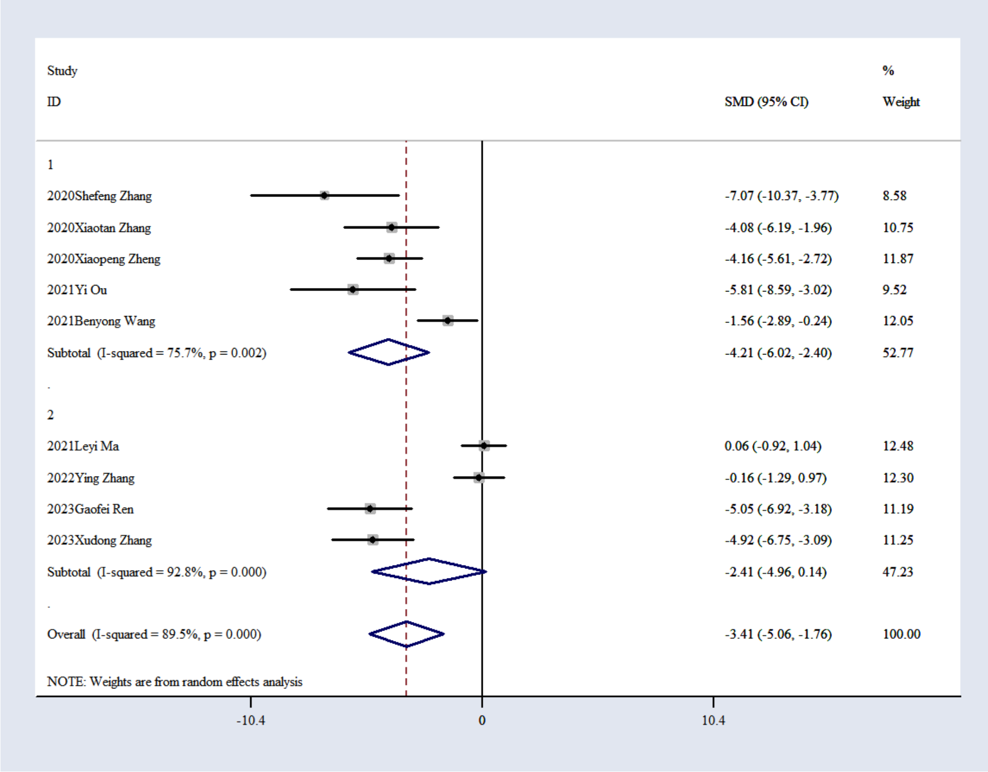

Supplementary Figure 12. Subgroup analysis of SCR based on modeling methodologies

Planta Med | DOI 10.1055/a-2615-8249 | © The Author(s).

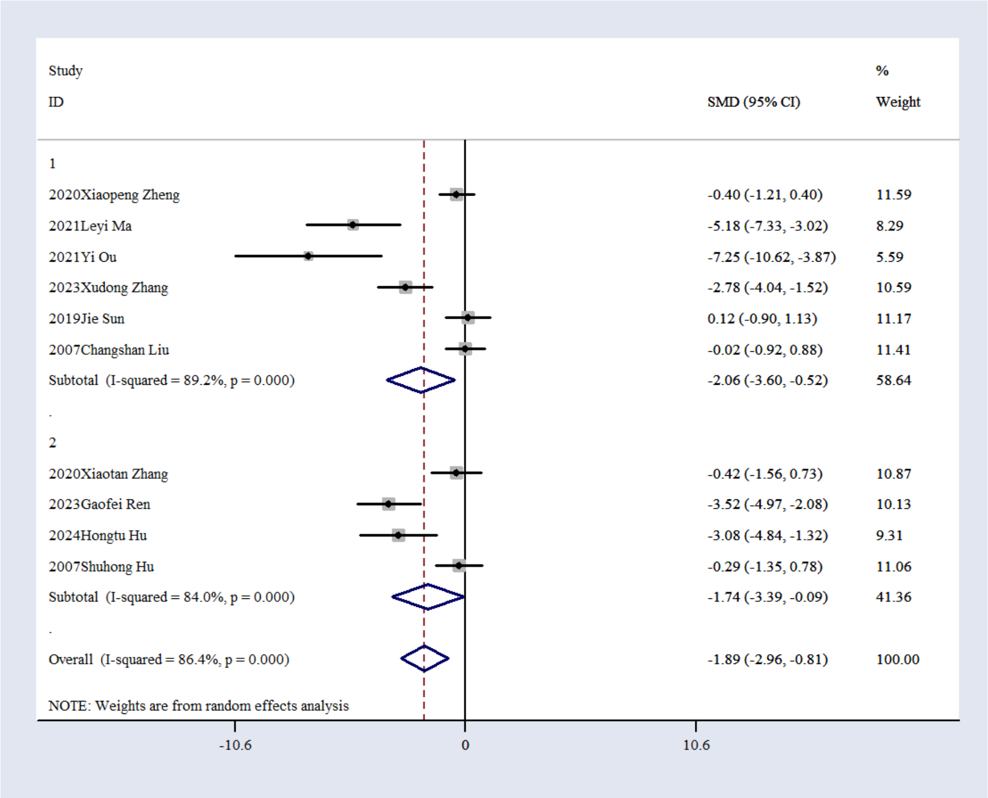

Supplementary Figure 13. Subgroup analysis of FBG based on intervention duration

Planta Med | DOI 10.1055/a-2615-8249 | © The Author(s).

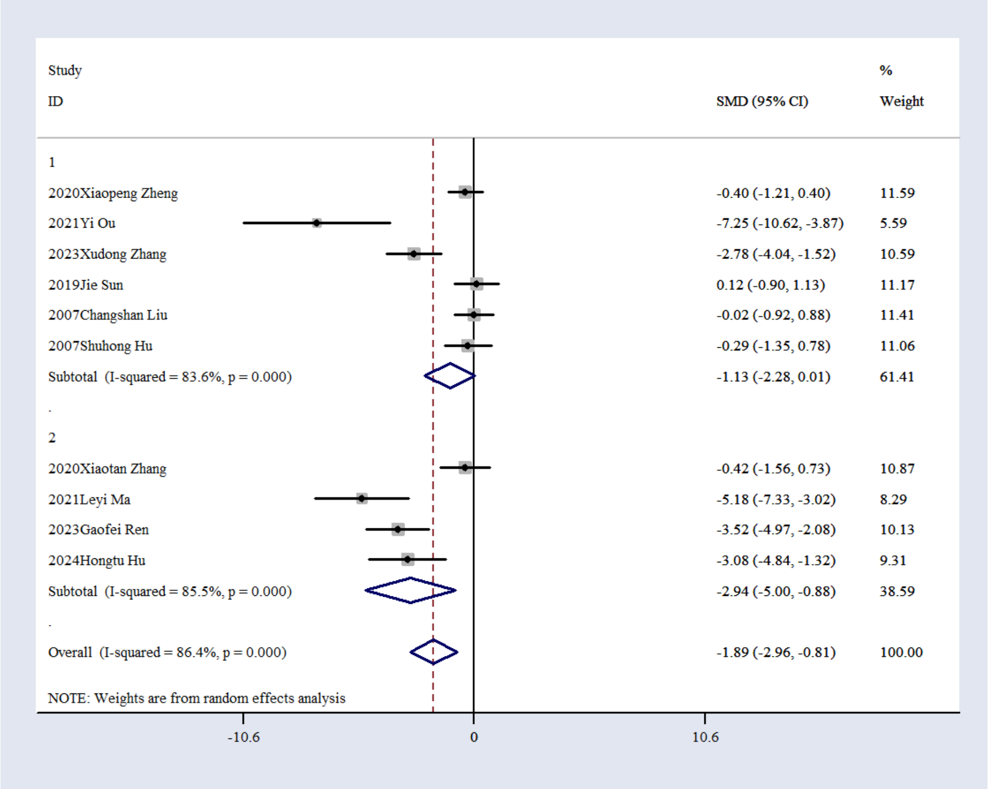

Supplementary Figure 14. Subgroup analysis of FBG based on species of animals

Planta Med | DOI 10.1055/a-2615-8249 | © The Author(s).

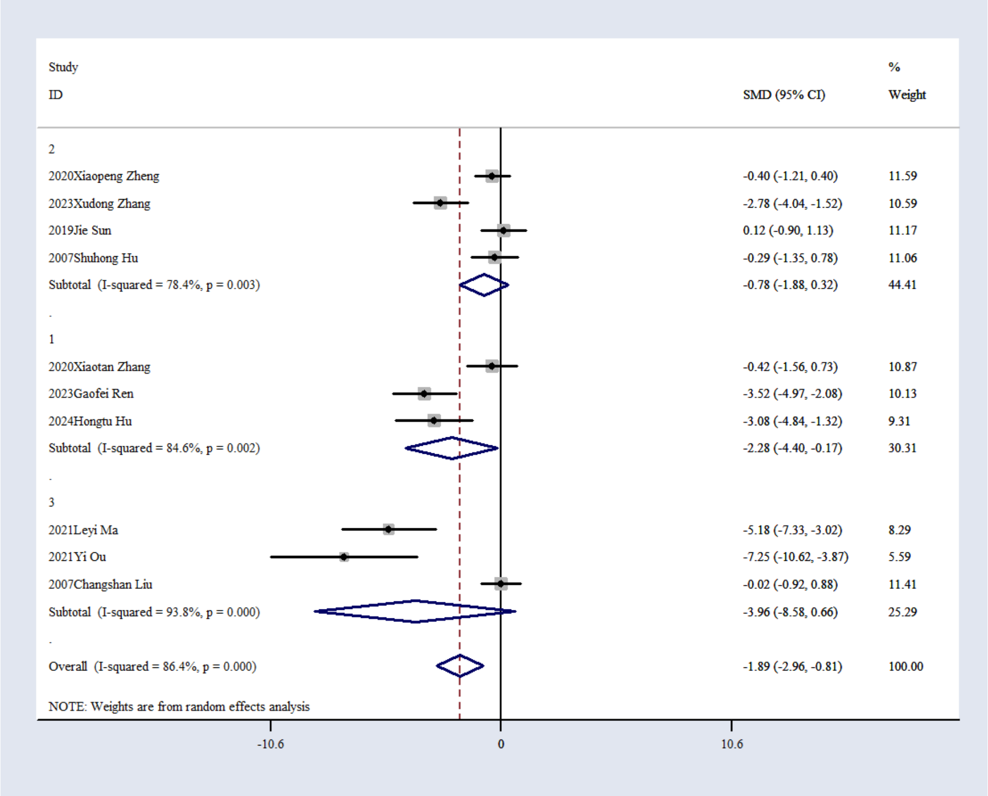

Supplementary Figure 15. Subgroup analysis of FBG based on dosage regimens of baicalin

Planta Med | DOI 10.1055/a-2615-8249 | © The Author(s).

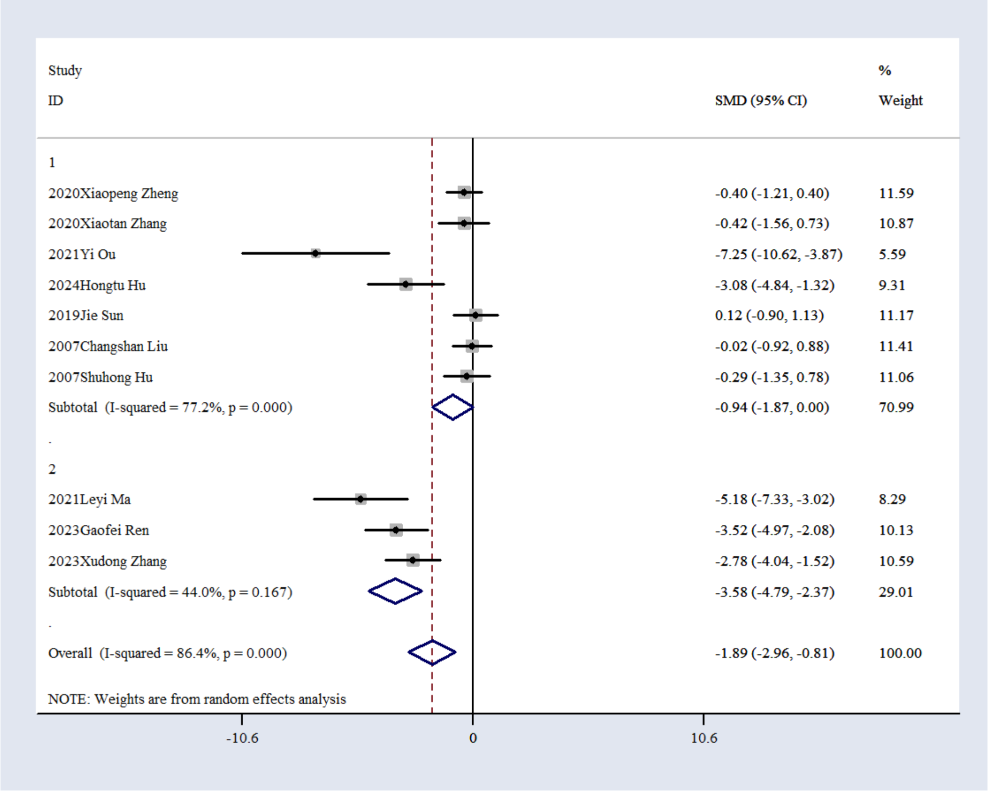

Supplementary Figure 16. Subgroup analysis of FBG based on modeling methodologies

Planta Med | DOI 10.1055/a-2615-8249 | © The Author(s).
